# Transcriptomic Analysis Corroborates the New Radial Model of the Mouse Pallial Amygdala

**DOI:** 10.3390/biom15081160

**Published:** 2025-08-13

**Authors:** Gloria Fernández, Lara López-González, Eduardo Pons-Fuster, Luis Puelles, Elena Garcia-Calero

**Affiliations:** Departamento de Anatomía Humana y Psicobiología, Facultad de Medicina, Universidad de Murcia e Instituto Murciano de Investigación Biosanitaria IMIB-Pascual Parrilla, 30120 Murcia, Spain; gloria.fernandez2@um.es (G.F.); eduardo.p.f@um.es (E.P.-F.); puelles@um.es (L.P.)

**Keywords:** amygdala, limbic system, mental disorders, transcriptomics

## Abstract

The mammalian amygdala is located in the temporal lobe of the telencephalon and plays a key role in limbic processing. Recently, our group proposed a radial morphological model to understand the glutamatergic (pallial) part of this nuclear complex in terms of separate progenitor domains. This model explains the amygdala region as consisting of several adjacent developmental radial progenitor units, disposing their distinct periventricular, intermediate, and superficial strata from the ventricle to the pial surface. It was expected that cell populations belonging to specific progenitor domains would present greater molecular similarity to each other than to neighboring developmental units. In this work, we aim to corroborate the existence of several radial domains in the pallial amygdala at the transcriptomic level. snRNAseq experiments in the amygdala of adult mice of both sexes indicated that at low resolution, the whole pallial amygdala was found to divide into two super-radial domains distinguished by differential expression of *Slc17a6* and *Slc17a7*; the former partly imitates molecularly the subpallial (output) amygdalar regions, whereas the rest of the pallial amygdala is molecularly more akin to the surrounding cortical areas. In addition, our snRNAseq transcriptomic analysis fully supports the postulated amygdalar radial model of four main radial domains.

## 1. Introduction

The amygdala is a still insufficiently understood nuclear complex (partly pallial and partly subpallial) located in the temporal pole of the mammalian telencephalon [1]. This neural structure is implicated in emotional processing and learning and is held to be an important hub in the connectome of the limbic system [2,3,4,5,6,7,8]. In this sense, its functional alteration is implicated in many psychiatric disorders such as depression, anxiety, autism, phobias, and post-traumatic stress [9]. Transcriptomic analysis of pallial (glutamatergic) and subpallial (GABAergic) amygdala in recent years has provided a richer view of amygdalar neuronal populations [10,11,12,13,14,15]. However, all these studies use conventional non-radial (oblique-section) topography as a morphological reference [16,17,18,19,20,21]. Conventional morphological study of the mammalian amygdala is based on coronal and horizontal sections in adult brains, but these section planes happen to be oblique to its intrinsic radial glial organization [19,22]. Adult models of intra-amygdalar nuclear relationships are accordingly inconsistent with the embryological notion of histogenetic radial domains [23]. The latter contemplates radially structured developmental units stratified from the ventricle to the pial surface. Comparative studies pose the added difficulty caused by varying degrees of species-specific morphogenetic deformation of the temporal pole region among different mammalian species [24]. This handicaps the establishment of homologies, particularly with non-mammalian species.

A morphological model based on how the brain is built during development will be a more appropriate reference framework for transcriptomic studies, allowing a much deeper molecular cell population analysis. We recently performed radial glia labeling and genoarchitectural studies, leading to the proposal of a *radial structural model* of the adult mouse *pallial amygdala* [22]. This model identifies four main radial amygdalar units: *anterior*, *lateral*, *basal*, and *posterior*, consistent with the intrinsic radial glial organization, and composed of periventricular, intermediate, and superficial strata in the radial dimension [22,25,26,27]. The prevision of this model, compared to previous ones, is that cells that share a domain, subdomain, or radial stratum are molecularly related to each other and therefore must form cellular clusters in transcriptomic studies. Glutamatergic amygdalar cells in a proposed radial unit/subunit must clusterize together in snRNAseq experiments if the radial pallial amygdalar model is right.

The issue directly addressed in the present contribution is whether single-cell transcriptomics performed on the adult mouse pallial amygdala corroborates and expands our radial model in terms of the four amygdalar radial units and the specific stratified nuclei proposed. Our attention centers on glutamatergic (pallial) neurons, considering that GABAergic cells in the pallial amygdala essentially reflect tangential migrations from external sources, best studied separately. We developed our transcriptomic analysis of pallial amygdala in the context of neighboring cortical and subpallial regions. Finally, our snRNAseq transcriptomic analysis at various resolutions, and the associated spatial expression studies of representative gene markers, fully support the postulated amygdalar radial model, implying a significant step forward in the understanding of the pallial amygdala in terms of neuronal cell types.

## 2. Materials and Methods

### 2.1. Animals

Adult mice (C57BL/6 strain, source University of Murcia) were sacrificed at the Animal Housekeeping Facility, CEIB, University of Murcia, following Spanish and European regulations: animals were first anesthetized with isoflurane at 4–5% and subsequently decapitated. Whole brains were extracted. Experimental protocols were approved by the University of Murcia Committee for Animal Experimental Ethics and CARM (Autonomous Community of the Region of Murcia; Approval Code, No. A13230704/Approval Date: 20 July 2023). Before sacrifice, the animals were kept in individual cages at the CEIB, under veterinary control.

### 2.2. Brain Extraction and Amygdalar Dissection

**Figure 1:**
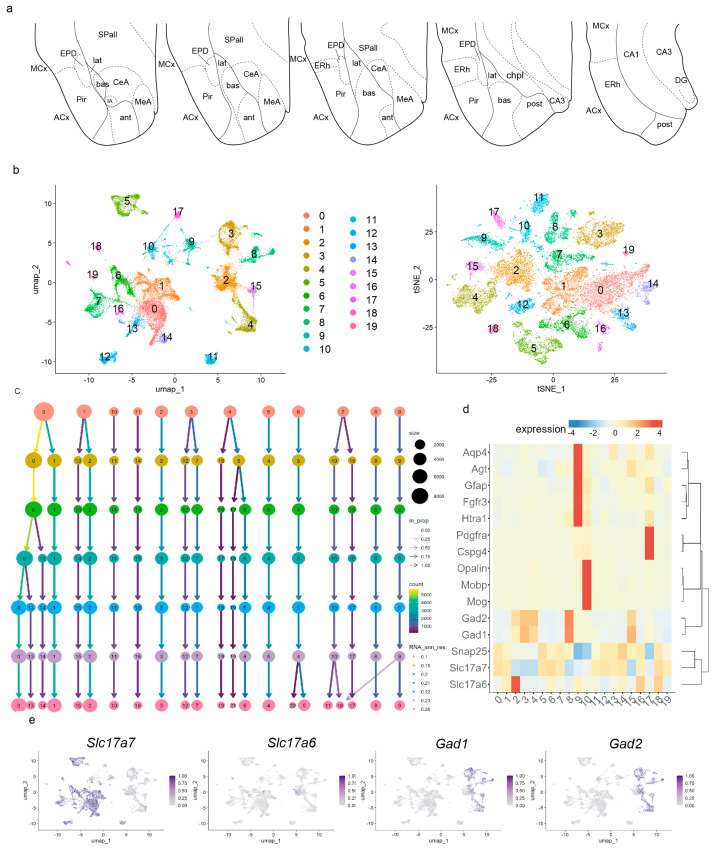
Pallial amygdala in the context of neighboring pallial and subpallial areas. (**a**) Schematic of the regions dissected in our four adult samples, including the pallial amygdala and other neighboring regions of the pallium and subpallium. (**b**) Uniform Manifold Approximation and Projection for Dimension Reduction (UMAP) and t-distributed stochastic neighbor embedding (t-SNE) plots of the adult Seurat object (merge of 4 samples; 31,848 nuclei), colored by clusters. (**c**) Clustree-0.5.1 package to visualize clustering of the adult Seurat object at resolutions 0.1, 0.15, 0.2, 0.21, 0.22, 0.23, and 0.25. (**d**) Heatmap of markers for cell type differentially expressed between clusters with gene hierarchical clustering. (**e**) UMAP plots showing expressions of *Slc17a7*, *Slc17a6*, *Gad*, and *Gad2* transcripts in the adult dataset. ACx, allocortical ring; ant, *anterior* radial domain; bas, *basal* radial domain; CA1, CA1 field; CA3, CA3 field; CeA, central amygdala; chpl, choroidal plexus; dentate gyrus, DG; ERh, entorhinal cortex; EPD, dorsal endopiriform nucleus; IA, intercalated amygdalar nucleus; lat, *lateral* radial domain; MCx, mesocortex; MeA, medial amygdala; Pir, piriform cortex; post, *posterior* radial domain; SPall, subpallium.

The amygdalar region was microdissected from entire whole brains using microsurgical knives under a dissection microscope (Leica S91, Leica Microsystems, Wetzlar, Germany) in ice-cold Sigma-Aldrich Dulbecco’s Phosphate Buffered Saline, without calcium and magnesium (Sigma-Aldrich cat# D8537, Merck Group, Darmstadt, Germany). For the amygdalar dissection, we established the separation of the fimbriae as the rostral coronal plane landmark, and the end of the telencephalic vesicle containing the caudal entorhinal cortex as the caudal limit, and dissected the area enclosing pallial/subpallial amygdala, neighboring cortical areas from the allo/mesocortex, and subpallial regions (represented in Figure 1a). The weight of the selected material was as follows: adult female batch 1 (AF1) 64.4 mg, adult female batch 2 (AF2) 44.4 mg, adult male batch 2 (AM2) 53.3 mg, and adult male batch 3 (AM3) 53.3 mg. The dissected tissue was frozen in liquid nitrogen and stored at −80 °C until used.

### 2.3. Single Nuclei Suspension

Frozen tissue samples were selected and placed in dry ice. Nuclei were isolated using the Minute™ single nucleus isolation kit designed for neuronal tissue/cells (Cat# BN-020, Invent Biotechnologies, Plymouth, MN, USA) with a modified version of the manufacturer’s protocol. The samples previously selected were briefly homogenized on ice, by ~60 pestle strokes, in a 1.5 mL tube with 200 μL buffer A (lysis buffer and RNAse inhibitor, Protector RNAse Inhibitor, Merck cat. 335399001, Merck Group, Darmstadt, Germany). Add 500 μL of buffer A and keep homogenizing (1–2 min in total). The homogenate was incubated on ice for 8 min and transferred to a filter-integrated collection tube. Following this, the tubes were centrifugated at 13,000× *g* for 30 s. The filtered samples were then centrifugated in a swinging bucket centrifuge at 600× *g* for 4 min. The resulting pellet was resuspended in 1 mL of PBS + 2% BSA (UltraPure™ BSA, ThermoFisher Scientific cat. AM2618, Waltham, MA, USA) and centrifugated again at 500× *g* for 5 min. The precipitate was resuspended in 150 µL of PBS + 2% BSA. The quality of the nuclei was checked under an inverted microscope (Leica mod. DMi8, Leica Microsystems, Wetzlar, Germany). If clumping or debris was high, nuclei suspension was filtered by a 40 μm cell strainer (Flowmi^®^ Cell Strainers; Merck cat. BAH136800040-50EA, Merck Group, Darmstadt, Germany). Concentration of nuclei in the final suspension was assessed by staining with Trypan Blue and counting using a hemocytometer.

### 2.4. cDNA Library Preparation, Sequencing, and Cell Ranger Processing

Nuclei suspensions from each adult sample was diluted to an appropriate concentration and used as input to the 10× Chromium platform, using a 10× Genomics Chromium controller (Molecular Biology Section-ACTI, University of Murcia) plus Chromium Single Cell products: Chromium Next GEM Single Cell 3′ Kit v3.1 (Cat#1000269), Chromium Next GEM Chip G Single Cell Kit (Cat# 1000127), Dual Index Kit TT Set A (GEX Library; Cat# 1000215, 10× Genomics, Leiden, The Netherlands). The four library constructions were performed according to the manufacturer’s instructions. The final libraries were quantified using a Qubit 4 fluorometer (Invitrogen, Waltham, MA, USA).

Premade-10X 3 prime Single Cell Transcriptome Libraries were sequenced by Novogene (Cambridge, UK) using Illumina Technology, Novaseq X Plus Series PE150, partial lane sequencing-with demultiplexing. Single-cell RNA sequencing data were processed using Cell Ranger v.6.0.0 (10× Genomics), a software suite designed for analyzing Chromium Single Cell data. Gene expression quantification was performed with “cellranger count”, which aligns reads to the reference genome using STAR [28], filters and corrects barcodes, and generates a gene–cell matrix. The output was further analyzed to assess sequencing quality and generate feature-barcode matrices for downstream analysis.

### 2.5. SnRNA-seq Data Quality Control Processing of Cell Datasets

Feature-barcode matrices from each adult sample were analyzed in R, using the Seurat-5.1.0 package [29] to generate individual Seurat objects: AF1, AF2, AM2, and AM3. Metadata corresponding to the sex, batch, sample, and stage was added to each object. Quality metrics were checked in each adult object. We used violin plots and scatter plots to check out the individual metric distributions (Features/Genes per nucleus; Counts/UMIs per nucleus; percentage of mitochondrial RNA per nucleus), correlations between metrics (genes vs. UMIs per nucleus), and library complexities in our four samples (Figure S1). The distributions of nFeature_RNA (number of genes detected) and nCount_RNA (number of UMIs) were concentrated within adequate ranges, suggesting that most nuclei had reasonable transcriptional complexity. The percentage of mitochondrial genes (percent.mt) was low in most cells, indicating that there was no massive cellular damage or high stress that could compromise the integrity of the nuclei. No excessively pronounced atypical tails or peaks were observed, indicating the absence of major contamination. In scatter plots of Genes vs. UMIs, the positive correlation between nFeature_RNA and nCount_RNA was clear, such that as the number of UMIs increases, more genes were detected. This is expected in high-quality scRNA-seq data. Most nuclei cluster homogeneously, without major deviations, indicating the absence of anomalous populations. The library complexity violin plots reflected transcriptional diversity relative to sequencing depth. It was observed that most nuclei showed adequate complexity, in line with expectations for an snRNA-seq experiment. In addition, to ensure high-quality samples, individual Seurat objects were processed independently to filter out droplets using the DoubletFinder package, version 2.0.6 [30].

Next, the Seurat objects of our 4 adult samples were merged into a single adult Seurat object. The total number of nuclei was 45,886 (3431, AF1; 15,857, AF2; 12,549, AM2; 14,049, AM3). We applied optimized quality filters to retain nuclei with these values: number of genes per cell minor than 2500, number of UMIs per cell major than 500 and minor than 5000, and percentage of mitochondrial RNA minor than 0.5%. The number of nuclei after quality filtering was 31,848. We checked again the quality of our adult Seurat object (Figure S2a; Features/Genes per nucleus; Counts/UMIs per nucleus; percentage of mitochondrial RNA per nucleus; genes vs. UMIs per nucleus; library complexity). Quality metrics indicated that the nuclei remaining after filtering have adequate characteristics (number of genes and UMIs, low mitochondrial percentage, and stable library complexity). This suggests that the data were reliable for further analysis (clustering, cell type identification, etc.) with minimal risk of bias due to poor quality or contamination.

### 2.6. SnRNA-seq Data Processing in Adult Seurat Object

The adult Seurat object (merge of 4 samples) was then normalized using the log-normalization method, scale factor of 10,000. To focus on genes with high nucleus-to-nucleus variation in our object, we selected 3000 highly variable features using the function “FindVariableFeatures” (selection.method = “vst”). The object was scaled (“ScaleData” function), and the regressed variables were: “percent.mt”, “nCount_RNA”, and “nFeature_RNA”. We performed an analysis of Principal Components (PCA) in the scaled data. An Elbow plot based on the percentage of variance from the first 50 PCs was analyzed, and after careful testing, we used the first 40 PCs for subsequent analysis: construction of a neighborhood graph (“FindNeighbors” function) and Louvain clustering (“FindClusters” function). For cell clustering, we tested different resolutions: 0.1, 0.15, 0.2, 0.21, 0.22, 0.23, and 0.25. The Clustree-0.5.1 package was used to visualize clustering at different resolutions. These results were reduced using Uniform Manifold Approximation and Projection for Dimension Reduction (UMAP) and t-distributed stochastic neighbor embedding (t-SNE) plots (Figure 1b,c); the number of clusters was 19. The optimal cluster resolution was 0.22, taking into consideration ulterior gene expression, cell type, and cell area annotations. Nuclei from the four original adult samples (AF1, AF2, AM2, and AM3) and both sexes were present and well mixed in all 19 clusters (Figure S2b).

Clusters were annotated based on the expression of well-established marker genes for cell type [13,14,31,32,33]: astroglia (*Fgfr3*, *Aqp4*, *Agt*, *Gfap*, *Htra1*); immature and mature oligodendroglia (*Cspg4*, *Pdgfra*, *Mog*, *Opalin*, *Mobp*); and neurons (*Snap25*), either excitatory *Slc17a6*, *Slc17a7*) or inhibitory (*Gad1*, *Gad2*) (Figure 1d). For cluster annotation of cell areas, we used the “FindAllMarkers” function (Wilcoxon Rank sum test by default) from Seurat-5.1.0 and the AGEA tool from AMBA (https://mouse.brain-map.org/agea (accessed on 24 June 2025)). Cell areas identified were: piriform cortex (Pir; *Nwd2*, *Igfn1*, *Trps1*, *Adcy8*, and *Cux2*); the lateral entorhinal area (ERhL; *Cdh20*, *Trps1*, *Adcy8*, *Pde11a*, *Cux2*, *Nwd2*, *Nr4a2*, *Satb2*); medial entorhinal cortex, pre/para-subiculum, subiculum, and CA1 field (ERhM/Pre-/Para-/Sub/CA1; *Dcn*, *Trpc4*, *Grp161*, *Pde11a*, *Tox*, *Cdh20*, *Adcy8*, *Cux2*, *Nr4a2*, and *Satb2*); CA3 field (CA3; *Tle4*, *Trpc4*, *Gpr161*, *Zfpm2*, and *Trps1*); dentate gyrus (DG; *Prox1*, *Trpc6* and *Zfpm2*); and pallial amygdala (APall; *Baiap3*, *Nova1*, *Zfpm2*, *Tox*, and *Rorb*). We constructed different heatmaps using the Shiny Cell package (normalized gene expression was average, log-transformed, and visualized in a plot: [34]. We visualized expression of the marker genes for cell type grouped by Seurat clusters (Figure 1d) and marker genes for cell area grouped by glutamatergic areas; genes were hierarchically clustered in these graphics.

### 2.7. SnRNA-seq Data Processing of Amygdalar Data

APall annotated clusters representing pallial amygdalar were subset, and the Seurat workflow was repeated with these parameters: 30 PCs for dimensionality and cluster resolution of 0.2. Visualization of clustering in UMAP and tSNE plots indicated the existence of GABAergic clusters in our data set (Figure S4a,b). After removing these GABAergic clusters, the total number of nuclei was 4717. We repeated the Seurat workflow (dimensionality 30 PCs) and tested different resolutions for our amygdalar dataset: 0.05, 0.1, 0.15, and 0.2. We visualized these data in the Clustree-0.5.1 package. The clustering results were visualized with UMAP and t-SNE plots, with the optimal resolution of 0.15. Nuclei from the four original adult samples (AF1, AF2, AM2, and AM3) and both sexes were present and well-mixed in all 4 clusters (Figure S4b). For cluster annotation of amygdalar clusters, we used the “FindAllMarkers” function (Wilcoxon Rank sum test by default) from Seurat-5.1.0 (S5) and the AGEA tool from AMBA. We constructed heatmaps for the amygdalar dataset using the Shiny Cell package mentioned previously [30]. We visualized expression of pallial amygdalar marker genes (*Tox*, *Cux2*, *Ddit4l*, *Rorb*, *Hgf*, *Satb1*, *Lypd1*, *Baiap3*, *Adarb2*, *Unc5d*, *Meis1*, *Nova1*, *Pbx3*, *Car12*, *Pde11a*, *Adcy8*, *Vgll3*, *Zbtb20*, *Reln*, *Cdh13*, *Ntng2*, *Nnat*, *Dkk3*, *Cyp26b1*, *Neurod6*, *Man1a*, *Etv1*, *Dcn*, *Sema5a*, *Meis2*, *Otof*, *Htr2c*, *Slc24a2*, *Dach1*, *Npsr1*, *Adamts2*, *Sema3e*, *Rspo2*, *Grm8*, *Zfp385b*) grouped by Seurat clusters at resolution 0.15. We constructed a heatmap for the *anterior* radial domain markers (*Baiap3*, *Adarb2*, *Unc5d*, *Sema5a*, *Meis1*, *Nova1*, *Pbx3*; clusters 1, 4, and 8 at cluster resolution 0.4). Markers for *posterior* radial domain (*Car12*, *Pde11a*, *Adcy8*, *Vgll3*, *Zbtb20*, *Reln*, *Cdh13*, *Rorb*, and *Dach1*; clusters 2 and 3 at resolution 0.2; clusters 0, 3, and 6 at resolution 0.4) were visualized in a similar heatmap. Markers for *lateral* and *basal* radial domains (*Tox*, *Cux2*, *Ddit4l*, *Rorb*, *Hgf*, *Satb1*, *Lypd1*, *Ntng2*, *Nnat*, *Dkk3*, *Cyp26b1*, *Neurod6*, *Man1a*, *Etv1*, *Dcn*, *Sema5a*, *Meis2*, *Otof*, *Htr2c*, *Slc24a2*, *Dach1*, *Npsr1*, *Adamts2*, *Sema3e*, *Rspo2*, *Grm8*, *Zfp385b*) were visualized in similar heatmaps at resolution 0.3 (cluster 3: bll, cluster 4:lat, cluster 5: blm). These heatmaps showed hierarchical gene clustering. UMAP plots for amygdalar pallial gene markers also used the Shiny Cell package.

### 2.8. Pallial Amygdalar Data Integration

We downloaded a mouse amygdalar dataset from Yu et al. [14]. We created a Seurat object with this data and checked the quality metrics (Figure S8a; Features/Genes per nucleus; Counts/UMIs per nucleus; percentage of mitochondrial RNA per nucleus). The samples in the metadata were from entire amygdala (“AMM1”, male, “AMM2”, male, “AMF1”, female, “AMF2”, female) or amygdalar regions as the central amygdala (“AMCEAf”, female, “AMCEAm”, male) or basolateral complex (“AMBLAm1”, male, “AMBLAm2”, male, “AMBLAf”, female). We subset the samples of whole amygdala (“AMM1”, “AMM2”, “AMF1”, “AMF2”). Cell types in the metadata were classified as astrocyte, pericyte, etc. We subset excitatory (glutamatergic) neurons from the main object (“ExN”). UMAP plots group/split by sample and sex indicated a well-mixed dataset (Figure S8b). We applied the Seurat workflow with these parameters: 20 PCs, and a low cluster resolution of 0.05. We visualized the clustering in a UMAP plot, obtaining two main clusters: one enriched in *Slc17a6* transcripts (cluster 0), and the other enriched in *Slc17a7* transcripts (cluster 1, Figure S8c,d). Due to previous results, we considered cluster 0 as the *anterior* radial domain, and cluster 1 must contain the *posterior*, *lateral*, and *basal* radial domains. We subclustered cluster 1, carefully checking different resolutions, and the optimal one was 0.12. We obtained a Seurat object composed of cluster 0 and subclusters 1_0, 1_1, 1_2 (Figure S8e). We checked the amygdalar radial markers in the four clusters and subclusters and annotated the radial domain that each cluster represented: cluster 0 was the *anterior* radial domain, cluster 1_0 was the *basal* radial domain, cluster 1_1 was the *lateral* radial domain, and cluster 1_2 was the *posterior* radial domain (Figure S8e). A heatmap with the amygdalar marker genes hierarchical clustering group by cluster and subcluster was generated as previously mentioned using the Shiny Cell package (Figure S8f).

We integrated our Seurat adult sample with the processed dataset from Yu et al. [14]. We tried different integrated methods: only merging both datasets, the “Harmony” integration method, and the canonical correlation analysis anchor-based algorithm (CCA; Figure S9a–c). This latter method produced a well-mixed integrated object (Figure S9c). We followed the Seurat workflow for the integrated object (30 PCs), and carefully studied different cluster resolutions (0.01, 0.05, 0.1, and 0.2). The optimal resolution was 0.1. We visualized our results in a UMAP plot (Figure S9e). We compared Seurat clusters in the integrated object, with radial domain clusters, area annotation, and amygdalar cell types in Yu et al. [14] dataset. We constructed UMAP plots for gene modules of the different radial domains. Genes expressed in every UMAP are indicated in the figures.

### 2.9. RNAscope In Situ Hybridization

Animals were anesthetized with isoflurane at 4–5% and subsequently decapitated. Whole brains were quickly removed and fresh frozen in O.C.T. compound and cut into 10 μL coronal sections using a cryostat (ThermoFisher Scientific HM525 NX, Waltham, MA, USA) and placed directly into slides (Epredia Superfrost Plus microscope slides cat: J1800AMNZ, ThermoFisher Scientific, Waltham, MA, USA). In situ hybridization was performed using the RNAscope Fluorescent Multiplex Kit (RNAscope^®^ Intro Pack for Multiplex Fluorescent Reagent Kit v2-Mm with TSA Vivid Dyes RNAscope^®^, Advanced Cell Diagnostics, Cat. 323280, Biotechne, Abingdon, UK) following the manufacturer’s instructions for fresh tissue. Slides containing the amygdala were used for staining. First, they were fixed for 1 h on 4 °C, 10% NBF, and dehydrated in a sequence of ethanol serial dilutions (50%, 70%, and 100%). After that, sections were briefly air-dried, and a hydrophobic barrier was drawn around the sections using a Pad Pen (Vector Labs, Oxford, UK). Slides were then treated with hydrogen peroxidase and protease III followed by the hybridization mixed target probe combination and incubated for 2 h in a humid chamber at 40 °C, for Mm-*Ntng2* (585811-C1), Mm-*Rspo2* (402001-C3), Mm-*Rorb* (444271-C4), Mm-*Cux2* (469551-C3), Mm-*Dach1* (412071-C3), Mm-*Pde11a* (481841-C1), Mm-*Sema3e* (449631-C2), Mm-*Baiap3* (1133511-C3), Mm-*Htr2c*-C1 (401001), Mm-*Etv1* (433281-C2), Mm-*Otof* (485671-C3), Mm-*Meis2* (436371-C4) and Mm-*Slc17a6* (319171-C2). Slices were incubated for 2 h in a humid chamber at 40 °C. To develop the signal, sections underwent a series of signal amplification and color development. Slides were then stained with DAPI and coverslipped.

### 2.10. Imaging

Digital microphotographs were acquired using a widefield microscope (Leica Thunder-imager) with a 20× objective and processed using a proprietary background subtraction (Leica ICC) that improves the results. The digital images were processed with ImageJ (NIH, https://imagej.net/ij/, (version V1.54k accessed on 24 June 2025)), Adobe Photoshop, (version Elements) and Adobe Illustrator (version CC) software (Adobe Systems MountainView, CA, USA).

### 2.11. AGEA Tool and In Situ Hybridization Figures from Allen Adult Mouse Brain Atlas

We used the AGEA data-mining function in the AMBA (https://mouse.brain-map.org/agea (accessed on 24 June 2025)) to find genes expressed non-ubiquitously in the mouse piriform cortex, lateral entorhinal cortex, medial entorhinal cortex, pre-/para-/subiculum, mesocortex, dorsal endopiriform nucleus, and pallial amygdala, discriminating between its four radial domains. A crosshair marker was placed in the mentioned glutamatergic regions recognized, and the “Find genes” tool was used to obtain a list of candidate genes of interest. We checked in detail the expression pattern of any promising candidates, selecting only those genes that seemed sufficiently discriminative, generally showing a sharp expression pattern. We downloaded in situ hybridization figures for the selected genes from the Allen Mouse Brain Atlas (RRID:SCR_017001; URLs: http://portal.brain-map.org (accessed on 24 June 2025)).

## 3. Results

### 3.1. Molecular Context of Pallial Amygdalar Neurons in the Telencephalic Temporal Pole

To verify pallial amygdalar radial subdivisions by transcriptomic analysis (snRNAseq, 10× Genomics), we first built a single-nuclei map of this region in the context of neighboring pallial and subpallial areas. We microdissected the amygdala region from the entire brains of adult mice (two males, two females; dissection schema in Figure 1a). The selected area included the pallial amygdala, schematized in our figures by its four radial subdivisions: *anterior* (ant), *lateral* (lat), *basal* (bas), and *posterior* (post) (Figure 1a) [22]. Neighboring pallial and subpallial territories present in the dissected tissue included parts of the allocortical ring, composed of the piriform cortex, entorhinal cortex, and hippocampal region (Figure 1a; ACx, Pir, ERh, CA1, CA3, DG). Adjacent parts of mesocortex were also included, containing, for instance, the dorsal endopiriform nucleus lying deep to piriform cortex (Figure 1a; MCx, EPD). Subpallial regions partially present in our material include the subpallial amygdala (medial, central, and intercalated amygdalar nuclei; Figure 1a; MeA, CeA, IA); striatal and pallidal areas were scarcely represented, if at all (Figure 1a; SPall). The hypothalamus was not included in our dissections.

After satisfactory high-quality control and filtering, we obtained a dataset of 31,848 high-quality single nuclei from our adult Seurat object (merge of four samples; Figures S1 and S2). For cluster resolution, we evaluated seven potential resolutions. We chose cluster resolution of 0.22 as the optimal one for differentiating the main cell types (astroglia, oligodendroglia, and neurons) and the main anatomical areas (pallial amygdala, subpallium, piriform cortex, lateral, and medial entorhinal cortex, hippocampal areas, mesocortical regions; see Uniform Manifold Approximation and Projection for Dimension Reduction, UMAP plot, and T-distributed Stochastic Neighbor Embedding, tSNE plot at cluster resolution 0.22 in Figure 1b; Clustree in Figure 1c). At cluster resolution 0.22, we observed 19 transcriptomic cell clusters, which were distributed among the four main cell types detected in our material by well-established markers (heatmap in Figure 1d) [13,14,31,32,33]: astroglia (*Fgfr3*, *Aqp4*, *Agt*, *Gfap*, *Htra1*; cluster 9); immature and mature oligodendroglia (*Cspg4*, *Pdgfra*, *Mog*, *Opalin*, *Mobp*; clusters 10 and 17); neurons were labelled with the pan-neural marker *Snap25* (clusters 0–8, 11–16, 18–19). We distinguished excitatory neurons expressing *Slc17a6* (cluster 2) and/or *Slc17a7* (clusters 0, 1, 5–7, 11–14, 16, 18, 19; Figure 1d; Figure 1e shows the UMAP plots for *Slc17a7* and *Slc17a6*). Inhibitory neurons express the markers *Gad1* and *Gad2* and correspond to clusters 3, 4, 8, and 15 (Figure 1d,e shows the UMAP plots for *Gad1* and *Gad2*).

**Figure 2:**
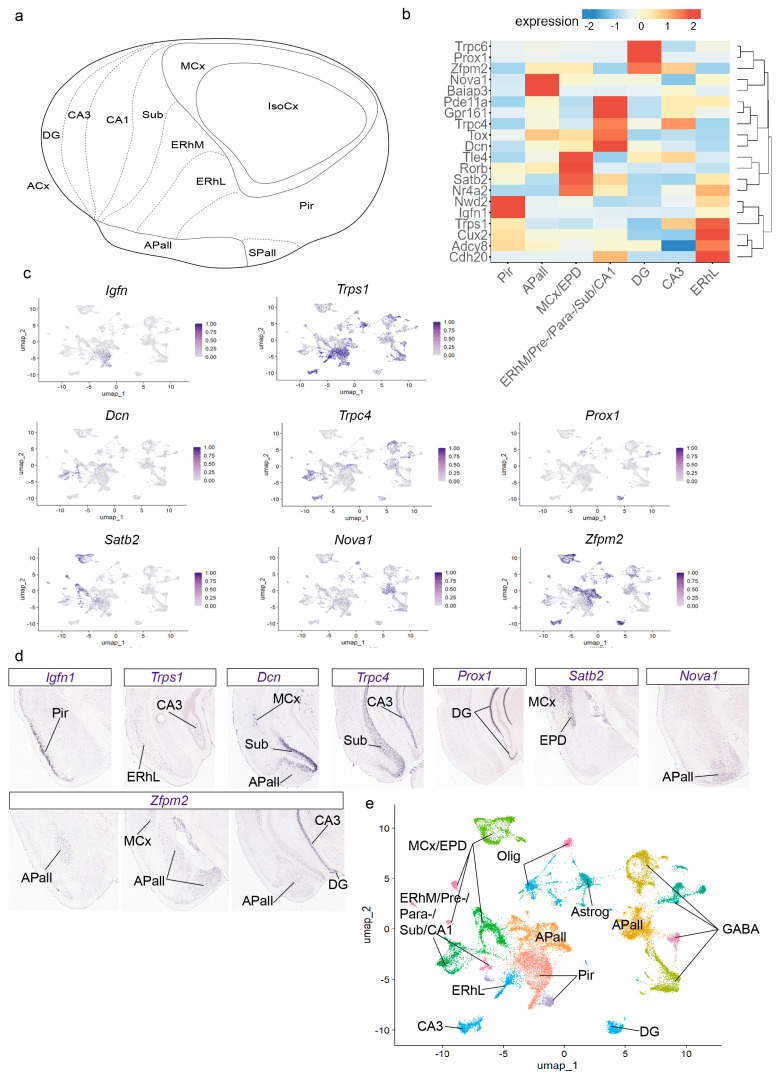
Cortical ring cell diversity and its relationship with pallial amygdala. (**a**) Flat cortical map representing the cortical ring model, with the pallial amygdala located outside the cortical area. (**b**) Heatmap of markers for cortical domains and amygdalar regions differentially expressed between clusters with gene hierarchical clustering. (**c**) UMAP plots showing expression of *Igfn1*, *Trps1*, *Dcn*, *Trpc4*, *Prox1*, *Satb2*, *Nova1*, and *Zfpm2* transcripts in the adult dataset. (**d**) In situ hybridization for *Igfn1*, *Trps1*, *Dcn*, *Trpc4*, *Prox1*, *Satb2*, *Nova1*, and *Zfpm2* downloaded from Allen Adult Mouse Brain Atlas (AMBA). Coronal plane. Scale bar 1200 µm. (**e**) UMAP plot of the adult object showing the different cell types, cortical areas, and pallial amygdala. ACx, allocortical ring; APall, pallial amygdala; CA1, CA1 field; CA3, CA3 field; DG, dentate gyrus; ERhL, lateral entorhinal cortex; ERhM, medial entorhinal cortex; IsoCx, isocortex; MCx, mesocortex; Pir, piriform cortex; SPall, subpallium.

To differentiate pallial amygdala from other glutamatergic regions, clusters enriched in the excitatory neuron markers *Slc17a7* and *Slc17a6* (clusters 0–2, 5–7, 11–14, 16, 18, and 19) were analyzed in the context of the cortical ring model (Figure 2a) [35,36]. In this model, the pallial amygdala lies outside the cortical area but is in close contact with the allocortical external ring, mainly at the levels of the posterior piriform cortex, entorhinal cortex, and the hippocampus (from subiculum to dentate gyrus) (Figure 2a). The perirhinal sector of the mesocortical ring lies beyond the entorhinal area, and thus is not in close contact with the pallial amygdala, but the periamygdalar dorsal endopiriform nucleus migrates from the depth of the perirhinal (mesocortical) area to the depth of the periamygdalar piriform cortex. Combinatorial genic profiles were analyzed to identify distinct cortical glutamatergic areas and pallial amygdala nuclei. We applied the “FindAllMarkers” function from Seurat-5.1.0 and the AGEA tool from Allen Mouse Brain Atlas (AMBA) to find these markers (Figure 2b). The piriform cortex (Pir) expressed *Igfn1*, *Trps1*, *Nwd2*, *Adcy8*, and *Cux2* (clusters 0 and 14 in Figure 1b; heatmap in Figure 2b; UMAP plots for *Igfn1*, *Trps1* in Figure 2c; *Igfn1*, *Trps1*, *Nwd2*, *Adcy8*, and *Cux2* expression analysis by in situ hybridization from AMBA in Figure 2d and Figure S3; Pir in Figure 2e). The lateral entorhinal area (ERhL) showed *Cdh20*, *Trps1*, *Adcy8*, *Pde11a*, *Cux2*, *Nwd2*, *Nr4a2*, and *Satb2* expression (cluster 13 in Figure 1b; heatmap in Figure 2b; UMAP plots for *Trps1* and *Satb2* in Figure 2c; *Cdh20*, *Trps1*, *Adcy8*, *Pde11a*, *Cux2*, *Nwd2*, *Satb2*, and *Nr4a2*, expression analysis by in situ hybridization from AMBA in Figure 2d and Figure S3; ERhL in Figure 2e). The markers *Dcn*, *Trpc4*, *Grp161*, *Pde11a*, *Tox*, *Cdh20*, *Cux2*, *Adcy8*, *Nr4a2*, and *Satb2* characterized the medial entorhinal area, pre-/para-subiculum, subiculum, and CA1 field (ERhM/Pre-/Para-/Sub/CA1; clusters 7, 16 in Figure 1b; heatmap in Figure 2b; UMAP plots for *Dcn*, *Trpc4*, and *Satb2* in Figure 2c; *Dcn*, *Trpc4*, *Grp161*, *Pde11a*, *Tox*, *Cdh20*, *Adcy8*, *Cux2*, *Satb2*, and *Nr4a2* expression analysis by in situ hybridization from AMBA in Figure 2d and Figure S3; ERhM/Pre-/Para-/Sub/CA1 in Figure 2e). The CA3 field was labelled with *Tle4*, *Trpc4*, *Gpr161*, *Zfpm2*, *Pd11a*, and *Trps1* markers, and the dentate gyrus (DG) by *Prox1*, *Trpc6*, *Tle4*, and *Zfpm2* (clusters 12 and 11, respectively, in Figure 1b; heatmap in Figure 2b; UMAP plots for *Trpc4*, *Prox1*, *Trps1*, and *Zfpm2* in Figure 2c; *Tle4*, *Trpc4*, *Gpr161*, *Zfpm2*, *Trps1*, *Prox1*, and *Trpc6* expression analysis by in situ hybridization from AMBA in Figure 2d and Figure S3; CA3 and DG in Figure 2e). The perirhinal mesocortex and the migrated dorsal endopiriform nucleus (MCx/EPD) expressed *Satb2*, *Nr4a2*, *Tle4*, *Rorb*, *Tox*, *Zfpm2*, and *Dcn* (clusters 5, 6, 18, and 19 in Figure 1b; heatmap in Figure 2b; UMAP plots for *Satb2*, *Zfpm2*, and *Dcn* in Figure 2c; *Satb2*, *Nr4a2*, *Tle4*, *Tox*, *Zfpm2*, and *Dcn* expression analysis by in situ hybridization from AMBA in Figure 2d and Figure S3; MCx/EPD in Figure 2e). The pallial amygdala (APall) expresses few genes that mark it as a whole (e.g., *Tbr1*, which is shared by all the pallium). The clusters observed via transcriptomics detect different gene combinations in different radial parts of the pallial amygdala, which collectively characterize the whole. Namely, *Zfpm2*, *Tox*, and *Rorb* are found in one cluster, while the *Nova1* and *Baiap3* markers appear in another distant cluster (clusters 1 and 2 in Figure 1b; compare APall in Figure 2e; UMAP plots for *Zfpm2* and *Nova1* in Figure 2c; *Nova1* and *Zfpm2* expression analysis by in situ hybridization from AMBA in Figure 2d). Cluster 2 is located molecularly close to GABAergic populations but is enriched in the glutamatergic marker *Slc17a6* (Figure 1b,e; APall in Figure 2e). Cluster 1 is molecularly adjacent to cortical areas and is enriched in the glutamatergic marker *Slc17a7*, as is the telencephalic cortex (Figure 1b,e; APall in Figure 2e). In addition, we observed that at low resolution, the piriform cortex, the amygdalar cluster 1, and the lateral entorhinal cortex cluster together (Clustree in Figure 1c). This result suggests a considerable molecular similarity between these three areas. Although the pallial amygdala lies outside the cortical field [35], it is located next to the piriform and lateral entorhinal cortex. Allocortical areas were not the focus of our study, but the present results also suggest some molecular similarity between medial entorhinal cortex and the hippocampal pre-/para-subiculum/subiculum and CA1 field, in contrast to the lateral entorhinal cortex, whose molecular profile resembles that of the piriform cortex, consistent with conclusions of Abellan et al. [37].

### 3.2. Glutamatergic Pallial Amygdala Populations Are Subdivided into Four Clusters Representing the Postulated Four Main Radial Domains

**Figure 3:**
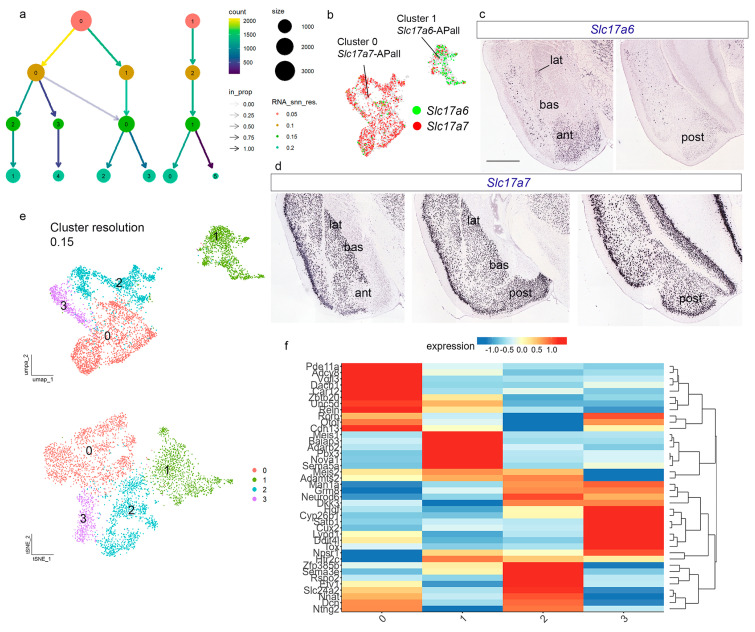
Transcriptomic analysis of the pallial amygdala. (**a**) Clustree-0.5.1 package to visualize clustering at resolutions: 0.05, 0.1, 0.15, 0.2, of the pallial amygdalar object. (**b**) UMAP for *Slc17a7* and *Slc17a6* transcripts in the pallial amygdalar dataset. (**c**) In situ hybridization for *Slc17a6* downloaded from AMBA. Scale bar 1200 µm. (**d**) In situ hybridization for *Slc17a7* downloaded from AMBA. Scale bar 1200 µm. (**e**) UMAP and tSNE plots for pallial amygdalar object at cluster resolution 0.15, colored by cluster. (**f**) Heatmap of markers for pallial amygdalar domains differentially expressed between clusters with gene hierarchical clustering. ant, *anterior* radial domain; APall, amygdala pallial; bas, *basal* radial domain; lat, *lateral* radial domain; post, *posterior* radial domain.

The data from the previous section showed the existence of two main pallial amygdalar clusters, identified as clusters 1 and 2 in Figure 1b, and as APall in Figure 2e. Next, we subclustered these two components out of the main Seurat object and removed GABAergic populations (Figure S4a,b). We obtained 4717 high-quality amygdalar pallial nuclei. To select a cluster resolution, we evaluated four potential resolutions: 0.05, 0.1, 0.15, and 0.2 (Clustree in Figure 3a). At the lowest value (0.05), we detected only the two main clusters mentioned above (named now 0 and 1; Figure 3a,b and Figure S4c). Cluster 0 was enriched in *Slc17a7* (*Slc17a7*-APall) transcripts, while cluster 1 was enriched in *Slc17a6* (*Slc17a6*-APall; Figure 3b). We checked the expression pattern of *Slc17a6* and *Slc17a7* genes in the pallial amygdala and identified *Slc17a6*-APall selectively at the *anterior* domain of the radial amygdalar model (Figure 3c), whereas the *Slc17a7*-APall subdivision was represented by the *lateral*, *basal* and *posterior* radial domains (ant, lat, bas, post; Figure 3d; in situ hybridization from AMBA) [22,27]. At cluster resolution 0.1, cluster 0 resulted subdivided into two new clusters, 0 and 1, and the former cluster 1 was renamed cluster 2 (UMAP plot in S4c). At cluster resolution 0.15, we observed four main clusters: the former cluster enriched in *Slc17a6*-APall was now cluster 1, while the former *Slc17a7*-APall cluster was subdivided now into clusters 0, 2, and 3 (UMAP and tSNE plots in Figure 3e). The four adult samples and both sexes were represented in the four clusters at resolution 0.15 (Figure S4d,e).

The postulated amygdalar radial model [22] proposes four main radial glutamatergic subdivisions in the pallial amygdala. We next checked whether the four clusters obtained at resolution 0.15 in our Seurat object relate to these four radial domains. We employed the “FindAllMarkers” function from Seurat-5.1.0 (Figure S5) to characterize precisely the different clusters, as well as the AGEA tool from AMBA and our previous results in Garcia-Calero et al. [22], to find markers for the different radial units or their component nuclei. We selected 40 genes expressed differentially among the four clusters and the diverse radial units (Figure 3f). The nomenclature we will use in the following text description of pallial amygdalar areas (as well as the figures) adopts a simplified graphic format, e.g., ‘*ant-i*’, corresponding to the abbreviation of the radial domain name (among *ant*; anterior; *post*; posterior; *lat*; lateral; *bas*; basal) separated by a *hyphen* from the abbreviation of the relevant radial stratum (-p; periventricular; -i; intermediate; -s; superficial).

Considering our results, we slightly changed the inner subdivision of the *basal* domain. Garcia-Calero et al. [22] already proposed two main radial subdomains of *bas* called *basolateral* (bl) and *basomedial* (bm), and it was thought that bm showed two mediolateral subdivisions (bmm, bml). In the present work, we keep the two main subdivisions *bl* and *bm*, but—consistent with specific data—subdivide bl, rather than bm, into lateral and medial subdivisions (*bll* and *blm*).

### 3.3. The Anterior Radial Domain

**Figure 4:**
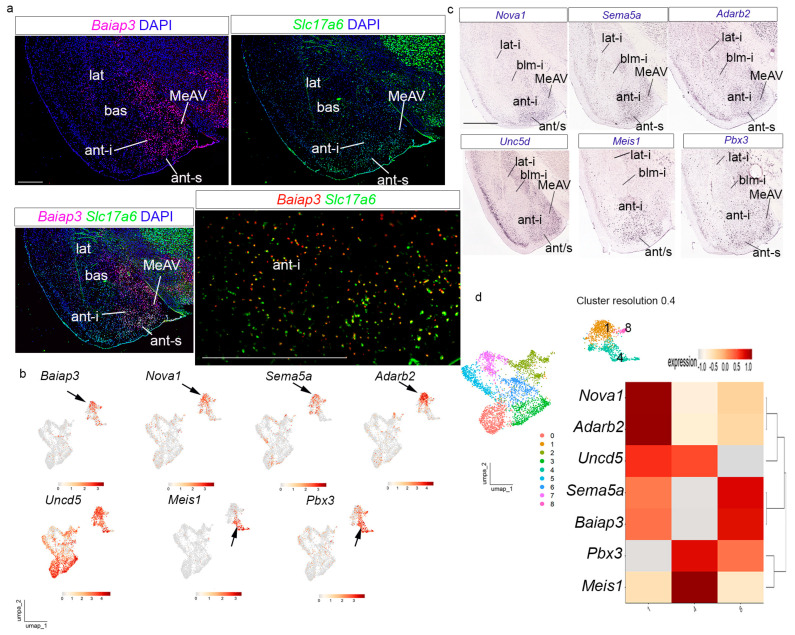
Transcriptomic data of the amygdalar *anterior* radial domain. (**a**) Multiplexed fluorescent in situ hybridization assay for *Baiap3* and *Slc17a6* in the amygdalar *anterior* radial domain. Combinations: *Baipa3*-DAPI, *Slc17a6*-DAPI, *Baiap3*-*Slc17a6*-DAPI, and *Baiap3*-*Slc17a6* magnification of ant-i area. Coronal plane. Scale bars 500 µm. (**b**) UMAP plots showing the expression of anterior radial markers: *Baiap3*, *Nova1*, *Sema5a*, *Adarb2*, *Uncd5*, *Meis1*, and *Pbx3.* (**c**) In situ hybridization for *Nova1*, *Sema5a*, *Adarb2*, *Unc5d*, *Meis1*, and *Pbx3* downloaded from AMBA. Scale bar 1200 µm. (**d**) UMAP plots of amygdalar dataset at resolution 0.4 colored by clusters (left) and heatmap gene markers for *anterior* radial domain at resolution 0.4, with hierarchical clustering of gene expression (right). ant-i, *anterior* radial domain, intermediate stratum; ant-s, *anterior* radial domain, superficial stratum; blm-i, *basomedio-medial* radial subdomain, intermediate stratum; lat-i, *lateral* radial domain, intermediate stratum.

We focused first on the *Slc17a6*-APall cluster (cluster 1 in Figure 3b,e), whose in situ expression was identified selectively at the *anterior* radial domain (ant; Figure 3c). This domain is constituted only by an intermediate stratum (ant-i) and a superficial stratum (ant-s), classically called, respectively, BMA (basomedial amygdaloid nucleus, anterior part) and ACo (anterior cortical amygdaloid nucleus), as identified by Paxinos and Franklin [21]; Garcia-Calero et al. [22] used a similar nomenclature. Garcia-Calero and Puelles [27] specifically described how the transient periventricular stratum of this radial domain disappears during development due to radial migration of its cells into the corresponding intermediate or superficial strata. We checked the expression of the chosen cluster 1 markers identified in Figure 3f: *Baiap3*, *Nova1*, *Sema5a*, *Adarb2*, *Unc5d*, *Meis1*, and *Pbx3*. The *Baiap3* and *Slc17a6* localized in both the ant-i and ant-s strata of the *anterior* radial domain; interestingly, the neighboring ventral medial amygdala also shows *Baiap3* and *Slc17a6* transcripts (ant-i, ant-s, MeAV; Figure 4a; note that Garcia-Calero and Puelles [27] reported a possible embryonic tangential migration of *Lhx9* cells from the *anterior* radial unit into the MeAV). The UMAP plot indicates a partly dispersed distribution of *Baiap3-*positive cells within the *anterior* radial cluster, indicating some molecular heterogeneity, perhaps due to the mixture of cells from different strata of the same radial unit (black arrow in Figure 4b). *Nova1*, *Sema5a*, *Adarb2*, and *Unc5d* genes are expressed similarly to *Baiap3*, with a partly heterogeneous distribution in the *anterior* radial domain and MeAV (Figure 4c; in situ hybridization from the AMBA). The corresponding UMAP plots for these genes showed a slightly less heterogeneous distribution, except for *Unc5d* (black arrows in Figure 4b). *Meis1* and *Pbx3* transcripts were localized mainly in the superficial stratum of the *anterior* radial domain and neighboring ventro-medial amygdala (ant-s; MeAV; Figure 4c; in situ hybridization from the AMBA); these transcripts, concentrated on superficial cells, were strongly represented in a particular heterogeneous strand of the corresponding UMAP plots (black arrows in Figure 4b).

This combination of gene expression patterns and UMAP studies indicated that the *anterior* radial domain (namely its intermediate and superficial strata) precisely corresponds anatomically to cluster 1. The expression of these genes indicates an inner molecular regionalization within this cluster, with a particular *Meis1* and *Pbx3*-expressing cell population located in the superficial stratum of the *anterior* domain. This regionalization was also observed at high cluster resolution (see, for example, the UMAP plot at resolution 0.4 and the gene distribution among the corresponding three clusters in Figure 4d). In addition, these results indicate that the snRNAseq technique was able to neatly distinguish at transcriptomic level the complete *anterior* radial domain—singularly devoid of a periventricular stratum—and its potentially migrated cells in the ventral medial amygdala (MeAV), showing marked molecular differences with the other three radial domains, as previously postulated by us [22,26,27].

### 3.4. The Posterior Radial Domain

**Figure 5:**
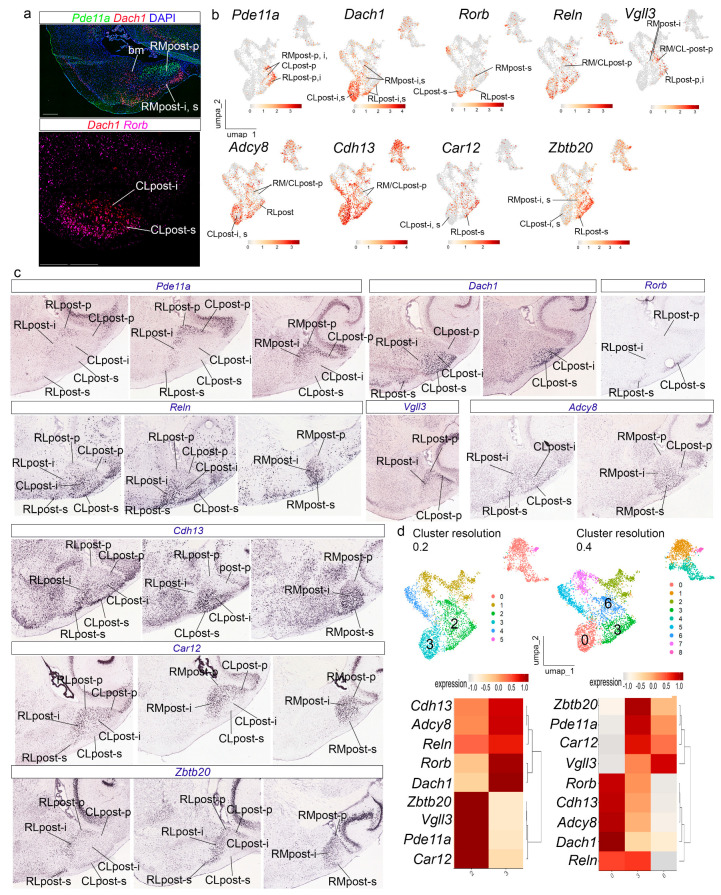
Transcriptomic data of the amygdalar *posterior* radial domain. (**a**) Multiplexed fluorescent in situ hybridization assay for *Pde11a*, *Dach1*, and *Rorb* in the amygdalar *posterior* radial domain. Coronal plane. Scale bars 500 µm. (**b**) UMAP plots showing the expression of posterior radial markers *Pde11a*, *Dach1*, *Rorb*, *Reln*, *Vgll3 Adcy8*, *Cdh13*, *Car12*, and *Zbtb20.* (**c**) In situ hybridization for *Pde11a*, *Dach1*, *Rorb*, *Reln*, *Vgll3 Adcy8*, *Cdh13*, *Car12* and *Zbtb20* downloaded from the AMBA. Sagittal plane. Scale bar 1200 µm. (**d**) UMAP plots of amygdalar dataset at resolutions 0.2 and 0.4, colored by clusters (up) and heatmap of gene markers for *posterior* radial domain at resolution 0.2 and 0.4, with hierarchical clustering of gene expression (down). bll-p, *basolatero-lateral* radial subdomain, periventricular stratum; blm-i, *basolatero-medial* radial subdomain, intermediate stratum; blm-p, *basolatero-medial* radial subdomain, periventricular stratum; bm, *basomedial* radial subdomain; CLpost-i, *cuadolateral posterior* radial subdomain, intermediate stratum; CLpost-p, *caudolateral posterior* radial subdomain, periventricular stratum; CLpost-s, *caudolateral posterior* radial subdomain, superficial stratum; lat-i, *lateral* radial domain, intermediate stratum; lat-p, *lateral* radial domain, periventricular stratum; post-i,s, *posterior* radial domain, intermediate and superficial strata; RMpost-i, *rostromedial posterior* radial domain, intermediate stratum; RMpost-p, *rostromedial posterior* radial domain, periventricular stratum; RMpost-s, *rostromedial posterior* radial domain, superficial stratum; RLpost-i, *rostrolateral posterior* radial subdomain, intermediate stratum; RLpost-p, *rostrolateral posterior* radial subdomain, periventricular stratum; RLpost-s, *rostrolateral posterior* radial subdomain, superficial stratum.

At resolution 0.15, the *Slc17a7*-APall cluster results subdivided into three main clusters (0, 2, and 3) expressing differentially a number of amygdalar gene markers (Figure 3e,f). Cluster 0 showed high expression of *Dach1*, *Pde11a*, *Adcy8*, *Car12*, *Reln*, *Zbtb20*, *Vgll3*, *Cdh13*, and *Rorb*. The combinatorial expression of these genes identified at cluster resolution 0.15 indicates that cluster 0 corresponds to the *posterior* radial domain (Figure 5) [22]. The periventricular stratum of this radial domain (post-p) corresponds to the amygdalo-hippocampal area (AHi) [21], while the intermediate and superficial strata (post-i, post-s) jointly compose the posteromedial cortical nucleus (PMCo) [21]. In addition, Garcia-Calero et al. [22] described three radial subdomains in this region: rostrolateral (RLpost), rostromedial (RMpost), and caudolateral (CLpost), which we tried to distinguish in our material. *Pde11a*, *Dach1*, and *Rorb* showed differential expression patterns in our in situ preparations, UMAP plots, and AMBA material, with abundant *Pde11a* transcripts in the whole RLpost, the periventricular stratum of the rest of the *posterior* radial domain, and the intermediate stratum in RMpost (RLpost-p, i, s; RMpost-p, i; CLpost-p; Figure 5a–c). *Dach1* labelled the intermediate and superficial strata in the *posterior* radial domain (RLpost-i,s; RMpost-i,s; CLpost-i,s), while *Rorb* transcripts were detected selectively in the corticoid plate or superficial stratum of the *posterior* domain, with a few transcripts in the intermediate stratum of the RLpost (RLpost-i,s; RMpost-s; CLpost-s; Figure 5a–c). UMAP plots for these three genes likewise showed differential expression patterns (Figure 5b).

*Reln* displayed a heterogeneous distribution of its transcripts in the *posterior* radial domain, labeling its periventricular (low expression), intermediate, and superficial strata (RLpost-p,i,s; RMpost-p,i,s; CLpost-p,i,s; low expression in RM/CLpost-p in UMAP plot in Figure 5b; in situ hybridization material from the AMBA in Figure 5c). *Vgll3* showed expression in the periventricular stratum of the *posterior* radial domain, and the intermediate stratum of RLpost and RMpost (RLpost-p, i; RMpost-p,i, CLpost-p; UMAP plot in Figure 5b; in situ hybridization material from the AMBA in Figure 5c). *Adcy8* and *Cdh13* transcripts also appeared in a heterogeneous pattern, with higher expression in the intermediate and superficial layers, and low expression in the periventricular stratum (RLpost-p,i,s; RMpost-p,i,s; CLpost-p,i,s; UMAP plots in Figure 5b; in situ hybridization material from the AMBA in Figure 5c); *Car12* and *Zbtb20* transcripts in the *posterior* radial domain, were represented by low expression in the superficial and intermediate strata of the CLpost (RLpost-p,i,s, RMpost-p,i,s; CLpost-i,s; UMAP plots in Figure 5b; in situ hybridization material from the AMBA in Figure 5c).

Increasing cluster resolution to 0.2 showed a partition of cluster 0 into two clusters, which represent, on one hand, the intermediate and superficial strata of the caudolateral subdomain and, on the other, the rest of the *posterior* radial domain (clusters 3 and 2, respectively; Figure 5d). Higher resolution 0.4 showed a certain degree of clustering by strata and by radial subdomains (Figure 5d).

### 3.5. The Lateral and Basal Radial Domains

**Figure 6:**
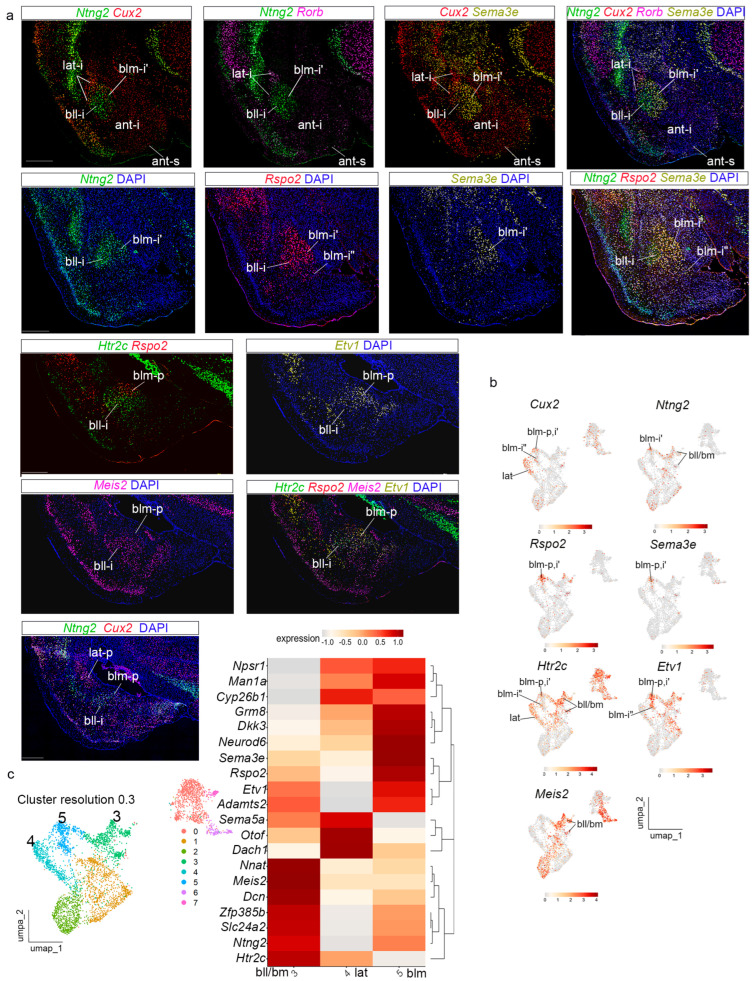
Transcriptomic data of the amygdalar *lateral* and *basal* radial domains. (**a**) Multiplexed fluorescent in situ hybridization assay for *Cux2*, *Ntng2*, *Rspo2*, *Sema3e*, *Htr2c*, *Etv1*, and *Meis2* in the amygdalar *lateral* and *basal* radial domains. Coronal plane. Scale bars 500 µm. (**b**) UMAP plots showing the expression of *lateral* and *basal* radial markers *Cux2*, *Ntng2*, *Rspo2*, *Sema3e*, *Htr2c*, *Etv1*, and *Meis2.* (**c**) UMAP plots of amygdalar dataset at resolution 0.3, colored by clusters. Heatmap of gene markers for *basal* radial domain at resolution 0.3, with hierarchical clustering of gene expression. bll, basolatero-lateral radial subdomain, intermediate stratum; blm-i’, blm-i”, basolatero-medial radial subdomain, intermediate stratum; blm-p, basolatero-medial radial subdomain, periventricular stratum; bm, basomedial radial subdomain; lat-i, lateral radial domain, intermediate stratum; lat-p, lateral radial domain, periventricular stratum.

We analyzed cluster 3 at resolution 0.15 (Figure 3e). Genes with high expression in this domain were: *Cux2*, *Rorb*, *Tox*, *Hgf*, *Cyp26b1*, *Lypd1*, *Ddit4l*, and *Satb1* (Figure 3f). Other genes also expressed in this cluster were *Neurod6*, *Grm8*, *Man1a*, *Dkk3*, *Npsr1*, and *Otof.* The expression of these genes indicated that cluster 3, at resolution 0.15, corresponds to the *lateral* radial domain (Figure 6, Figures S6 and S7). *Cux2* and *Rorb* were analyzed together with markers of the *basal* radial domain (*Rspo2*, *Sema3e*, *Ntng2*) to differentiate the *lateral* and *basal* radial domains (Figure 6a). *Cux2* and *Rorb* labelled the intermediate and periventricular strata of the *lateral* radial domain (lat-i; lat-p; Figure 6a). UMAP plots for *Cux2* showed more heterogeneous distribution of positive nuclei than *Rorb* UMAP (Figure 5b and Figure 6b). Other genes such as *Tox*, *Hgf*, *Satb1*, *Lypd1*, *Ddit4l*, and *Cyp26b1* showed similar expression patterns throughout the radial domain; *Otof* was expressed mainly in the periventricular stratum (see in situ hybridization material from AMBA material and UMAP plots; Figures S6 and S7). The superficial stratum of the *lateral* radial domain (lat-s), a subpial corticoid portion classically identified as ‘cortex-amygdala transition area’ (CxA), was not specifically detected in our material, possibly due to molecular similarity with the piriform cortex.

Next, we analyzed cluster 2 at resolution 0.15 (Figure 3e,f). Genes strongly expressed in this domain included *Sema3e*, *Rspo2*, *Ntng2*, *Etv1*, *Adamts2*, *Slc24a2*, *Nnat*, *Dcn*, *Neurod6*, *Man1a*, *Dkk3*, *Zfp385b*, and *Grm8.* In addition, we checked the expression of *Meis2*, *Htrc2*, and *Npsr1*, also expressed in this cluster. The transcripts of these genes indicated that cluster 2 represents the *basal* radial domain of Garcia-Calero et al. [22], which was subdivided into *basolateral* and *basomedial* radial subdomains (bl and bm). Present results support these subdivisions and further suggest a partition of bl into lateral and medial components (bll, blm). We distinguish now an internal subdivision in the intermediate stratum of blm, namely deep and superficial subregions (blm-i’; blm-i”). *Ntng2* and *Man1a* were expressed in the intermediate and periventricular strata of the whole basolateral subdomain (bll + blm; Figure 6a; see also in situ hybridization image from the AMBA in S7A; UMAP plots for these genes in Figure 6b and Figure S7b). In contrast, *Rspo2* transcripts mainly marked the medial subdivision of the intermediate and periventricular basolateral strata (blm-i’, blm-p), which is also selectively labelled by *Sema3e*, *Adamts2*, and *Npsr1* (Figure 6a and Figure S7a; see UMAP plots in Figure 6b and Figure S7b)*. Rspo2* transcripts appeared in the superficial blm-i (blm-i”; Figure 6a). *Neurod6* and *Dkk3* labelled selectively this superficial stratum of blm (blm-i”; Figure S7a; See UMAP plots in Figure S7b). In contrast, *Meis2* labelled differentially the intermediate and periventricular strata of the lateral subdivision in the basolateral subdomain (bll-i; Figure 6a; see UMAP plots in Figure 6b). *Htr2c* showed high expression in the whole bll subdomain (expression pattern and UMAP; Figure 6a,b). *Etv1* appeared expressed in the whole intermediate stratum of the basolateral division but showed more numerous transcripts in its medial subdomain (blm-p; Figure 6a and UMAP plot in Figure 6b). *Nnat* and *Dcn* labelled the basomedial subdivision of the basal radial domain (bm), in addition to other basal structures (Figure S7a and UMAP in Figure S7b). Other genes, such as *Slc24a2*, *Zfp385b*, and *Grm8*, showed more heterogeneous expression in the *basal* radial domain (Figure S7a; UMAP plots in Figure S7b). *Cyp26b1* showed low amygdalar expression in our snRNAseq experiment (Figure S7a and UMAP plot in Figure S7b). At a higher cluster resolution of 0.3, the *basal* domain appears subdivided into two clusters (Figure 6c). The heatmap of these genes indicated that the basal domain is subdivided mainly into the described bll + bm and blm radial areas. The bm division of the basal radial unit was not detected specifically among the different clusters obtained, though we clearly can distinguish this partition histologically in our preparations (bm; Figure 6a andFigure S7a). Other negative data refer to the fact that the subpial corticoid plates corresponding to the basal unit (PLCo and caudal CxA) were not specifically detected, possibly again due to molecular similarity with the piriform cortex.

### 3.6. Integration with Another Mouse Pallial Amygdalar Dataset Corroborates Our Transcriptomic Analysis in the Pallial Amygdala

To check the robustness of our present analysis, we integrated our adult amygdalar transcriptomic data with a recently published mouse amygdalar dataset of similar facture [14]. Before integration, we processed independently the results from these authors. We subsetted the excitatory neurons in the four samples of the published dataset that contain the whole amygdala (two males, two females; “sample” and “cell type” in the metadata). We obtained 3380 high-quality single nuclei in these samples, and male and female nuclei were well mixed (metrics in Figure S8a,b). At low cluster resolution (0.05), we again detected the existence of two main clusters, one enriched in *Slc17a6* (cluster 0), and the other enriched in *Slc17a7* (cluster 1), corroborating our own results of two such main clusters in the pallial amygdala (Figure S8c,d). Subclustering cluster 1 (named *Slc17a7*-APall one) and carefully selecting the subclustering resolution criteria indicated that at subcluster resolution 0.12, cluster 1 subdivides into three subclusters: 1_0, 1_1, and 1_2 (Figure S8e), while cluster 0 remains unitary. Analysis of radial domain markers in these two clusters and the three subclusters of cluster 1 (Figure S8f) indicated that cluster 0 represents the *anterior* radial domain (markers: *Baiap3*, *Nova1*, *Sema5a*, *Adarb2*, *Unc5d*, *Meis1*, and *Pbx3*), subcluster 1_0 corresponds to the *basal* radial domain (markers: *Sema3e*, *Rspo2*, *Ntng2*, *Etv1*, *Adamts2*, *Slc24a2*, *Nnat*, *Dcn*, *Neurod6*, *Man1a*, *Dkk3*, *Zfp385b*, *Grm8*, *Meis2*, *Htrc2*, and *Npsr1*), subcluster 1_1 corresponds to the *lateral* radial domain (markers *Cux2*, *Rorb*, *Tox*, *Hgf*, *Lypd1*, *Dditl4*, and *Cyp26b1*), and subcluster 1_3 represents the *posterior* radial domain (markers: *Dach1*, *Pde11a*, *Adcy8*, *Car12*, *Reln*, *Zbtb20*, *Vgll3*, *Cdh13*, and *Rorb*; Figure S8f).

**Figure 7:**
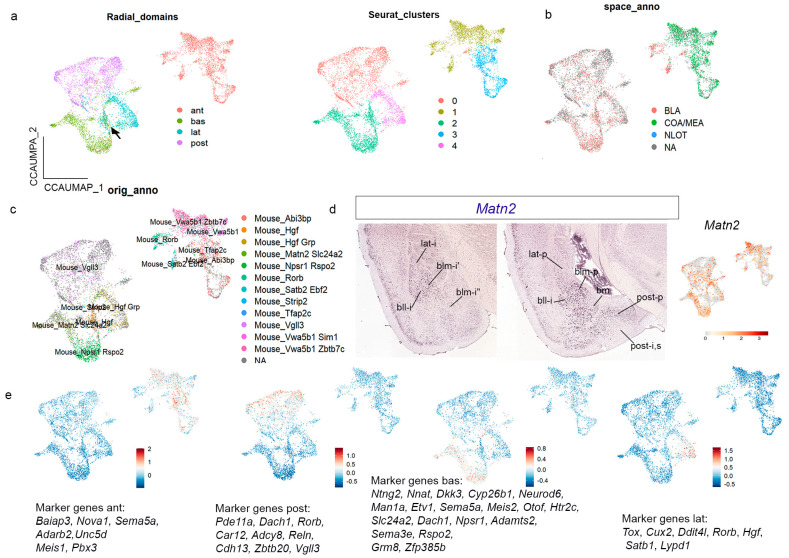
Pallial amygdalar integrated dataset to corroborate transcriptomic evidence of radial domains. (**a**) UMAP plots of the amygdalar integrated datasets from our own adults and Yu et al. [14] mouse transcriptomic analysis, colored by clusters and radial domains. (**b**) UMAP plot of the amygdalar integrated dataset colored by amygdalar areas annotated in Yu et al. [14] dataset. (**c**) UMAP plot of the amygdalar integrated dataset colored by amygdalar cell types annotated in Yu et al. [14] dataset. (**d**) *Matn2* expression pattern in pallial amygdala downloaded from AMBA (right; coronal plane; scale bar 1200 µm) and UMAP for the expression of this gene in the amygdalar integrated dataset. (**e**) UMAP plots of the amygdalar integrated datasets colored by group of gene expression indicated in each UMAP.

Next, we integrated our adult sample with this processed dataset from Yu et al. [14], testing different integrative methods (Figure S9a–c), and chose the canonical anchor-based correlation-analysis algorithm (CCA), which produced well-mixed sample populations (Figure S9c). We observed that in our integrated object, the cell nuclei from each of the four radial domains co-clustered together separately, except for a group of nuclei in between the lateral and basal domains (black arrow in Figure 7a). At cluster resolution 0.1, we detected five clusters that largely coincide with the aforementioned amygdalar radial domains, except that the *anterior* radial domain appears subdivided into two clusters in the integrated object (Figure 7a).

We analyzed the annotation for amygdalar cell areas in the Yu et al. [14] dataset in our integrated object (Figure 7b). It was observed that the *anterior* radial domain of our radial model was described in Yu et al. [14] as ACo + MeA. We agree that ACo (ant-s) and glutamatergic cells within the medial amygdala are included in our cluster for the *anterior* radial domain, but we also add the corresponding ant-i stratum, classically known as BMA. In addition, our clusters for the *lateral*, *basal*, and *posterior* radial domains were jointly identified as ‘the BLA area’ in the Yu et al. [14] dataset. Our radial model predicts a larger number of subdivisions in the amygdala, which is supported by the integrated transcriptomic analysis.

Next, we studied the cell types annotated by Yu et al. [14] in relation to gene expression in the integrated object (Figure 7c). In the *anterior* radial cluster, Yu et al. [14] annotated six cell types (*Vwa5b1/Zbtb7c*; *Vwa5b1/Sim1*; *Rorb*; *Tfap2c*; *Abi3bp*; and *Satb2/Ebf2*). We checked the expression of these genes in the AMBA material: *Zbtb7c* was clearly enriched in the *anterior* radial domain, while *Vwa5b1*, *Tfap2c*, *Abi3bp*, and *Ebf2* showed slight expression in this area. *Sim1* was studied previously in Garcia-Calero et al. [25] and represents a hypothalamic cell population migrated tangentially to the amygdalar region. *Rorb* and *Satb2* were analyzed in the present report. Yu et al. [14] characterized the *posterior* radial cluster as a single *Vgll3*-positive cell population. The *lateral* radial cluster also contained one distinct cell type (*Hgf*). The *basal* domain contained two cell populations (*Matn2/Slc24a2*; *Npsr1/Rspo2*). A *Hgf/Grp*-positive cell population lay at the boundary between the *basal* and *lateral* domains. *Matn2* labels lat-s, bll-p,i,s, bm-p,i,s, and blm-i, and shows dispersed expression in the *posterior* radial domain (post-p,i,s; Figure 7d). Its UMAP confirmed these results and their correspondence with the amygdalar pallial model. *Vgll3*, *Hgf*, *Slc24a2*, *Npsr1*, and *Rspo2* were also analyzed by us, and the combined expression pattern was found useful for visualizing the amygdalar radial domains. Finally, we further validated these results by constructing UMAP plots for gene modules (list of genes for every radial domain; list of gene markers indicated in the figures; Figure 7e).

## 4. Discussion

The amygdalar radial model [22] proposed the division of glutamatergic neuronal populations in the mouse pallial amygdala in four main radial (progenitor) domains (*anterior*, *posterior*, *lateral*, and *basal*), subdivided into periventricular, intermediate, and superficial strata. Successive neuronal types originating within a particular progenitor domain potentially express some shared molecular properties due to common inheritance from the ventricular matrix cells. The present report reflects our interest in checking this assumption by means of transcriptomic analysis of the molecular diversity or clustering pattern and relative positions along the planar and radial dimensions (radial units or parallel strata) of the glutamatergic (pallial) neuronal types examined at the level of single nuclear RNA transcripts.

In the present work on mouse pallial amygdalar neurons, we examined various levels of cluster resolution (Clustree function), both in our adult sample and after integrating our data with the similar mouse amygdalar dataset of Yu et al. [14]. The observed clusters and/or subclusters were checked and satisfactorily correlated with pallial amygdala parts according to the radial amygdalar model. The results amply supported consistency between the main cell clusters obtained and the four principal amygdalar radial domains, thus corroborating the assumptions and structural postulates of our radial model. In addition, we reprocessed Yu et al.’s [14] data on the amygdala and integrated them with our results. Both datasets were mutually consistent as regards clustering properties, as well as with the more precise anatomic resolution allowed by our radial (natural) interpretive model.

### 4.1. Two Big Super-Radial Domains Conform the Pallial Amygdala

In our Seurat object containing different cell types belonging to both cortical and amygdalar pallial areas and some neighboring subpallial regions (astroglia, oligodendroglia, and neurons), the pallial amygdala (labelled in the UMAP plots as APall) was reproducibly composed (also with the Yu et al. [14] data) by two main clusters. One of these clusters was enriched in the gene *Slc17a6*. This cluster is located in UMAP plots close to subpallial GABAergic components and appears relatively separated from the other amygdalar cells. The expression patterns of representative markers indicated that this cluster corresponds strictly to the *anterior* radial domain of Garcia-Calero et al. [22] or the classical BMA and ACo nuclei (basomedial anterior and anterior corticoid nuclei; see next section). The other major cluster in our dataset corresponds to the set of *posterior*, *lateral*, and *basal* radial domains of the radial model, which comprise all the remaining amygdalar nuclei, differentially enriched in the gene *Slc17a7*, which is widely expressed in the cerebral cortex. This cluster appears molecularly close to glutamatergic cortical regions, such as the piriform cortex.

Therefore, there is a sharp differential molecular characterization of two main radial super-domains in the glutamatergic amygdalar population, detected on the basis of the *Slc17a6* and *Slc17a7* markers. This division coincides with the developmental and anatomical distinction of the *anterior* radial unit, which actually develops next to the amygdalar subpallium, from the set of *lateral*, *basal* and *posterior* radial units, which anatomically neighbor the cortical pallium (note the *lateral* domain also contacts the striatum with its periventricular stratum, but molecularly they are not close to each other).

### 4.2. The Peculiarity of the Anterior Radial Domain

García-Calero et al. [22] already observed that the molecular profile of the *anterior* radial domain was different from that of the other radial domains described in the amygdala. Reviewing the neurogenesis of this region, it was further observed that its neurons were produced much earlier than those in neighboring radial complexes such as the *lateral* or *basal* domains [26,27]. The results of the current transcriptomic study clearly confirm the essential difference of this *anterior* radial domain, which appears as an isolated cluster, close in UMAP plots to GABAergic regions and selectively expressing *Slc17a6*, a marker with low expression in cortical regions. On the other hand, *anterior* domain expression data presented now or previously indicate aspects of molecular similarity of this domain with neighboring ventral regions of the medial amygdala (MeAV). This result is important, not only for the description of the molecular peculiarity of this domain, but also for the functional implications that may be entailed. It means the *anterior* radial amygdalar unit associated with the ventral medial amygdala is a subregion of the telencephalon that is molecularly less similar to the cortex than the rest of the amygdala. This singularity, representing a modified radial pallial column, lies between the caudal aspect of the telencephalic subpallium and the rest of the amygdalar pallium. As we have already stated, this *anterior* amygdala region exhibits earlier neurogenesis, and, therefore, its circuitry may also emerge earlier than in other amygdalar radial domains, perhaps acquiring particular functional relevance.

### 4.3. Transcriptomic Data on the Basal, Posterior, and Lateral Radial Units

We subtly modified the subdivisions of the *basal* radial domain, considering the transcriptomic results obtained and some corroborating in situ data. This radial domain emerges next to the *lateral* and *posterior* domains at a clustering resolution of 0.15. However, understanding its subdivisions apparently implies small adjustments to the model. In Garcia-Calero et al. [22], the basal domain was divided into *basolateral* and *basomedial* subdomains. The *basolateral* subdomain was, in turn, subdivided here transcriptomically into two parts (bll, blm), whereas we had previously left it unitary and partitioned instead the basomedial component on the basis of descriptive gene data [22] (Garcia-Calero et al. 2020). Various gene markers considered here support the bll/blm distinction within the *basolateral* subdomain, also with increased resolution. In contrast, the *basomedial* domain was included in the *basolateral* subdivision in our transcriptomic analysis, although we continue to differentiate it histologically from other markers, such as, e.g., *Azin2*.

Garcia-Calero et al. [22] recognized the *posterior* radial domain as the one lying close to the hippocampal region. It appeared subdivided into molecularly distinguishable rostrolateral, rostromedial, and caudolateral radial subdomains. In the current transcriptomic data (our own results), we detected the *posterior* radial domain as a whole at 0.15 resolution (see Figure 5). Increasing clustering resolution showed a certain degree of clustering into its predicted radial subdomains and strata.

Finally, the *lateral* radial domain is detected clearly in our material, with the exception of its corticoid (superficial) plate (classical CxA). Increasing cluster resolution did not divide this region, indicating a more homogeneous domain that was postulated before [21] largely in relation to connectivity patterns (reviewed in LeDoux [3]).

## 5. Conclusions

We think that our radial amygdalar model—now certainly corroborated in its main lines—is helpful in the interpretation of local neuronal typology and general genoarchitectural understanding of the amygdala within its pallial topology. Ulterior advances are expected on the discovery of functional computational algorithms of the diverse amygdalar cell assemblies and their participation in various limbic circuits, as well as their relationship with mental disorders. The corroborated radial model makes possible a novel recapitulative scenario of amygdalar modules (the radial units), as their intrinsic and extrinsic connections, as well as correlative functions, can now be explored. This requires visualizing the whole pallial amygdala complex, known to be crucially involved in the limbic system, as subdivided into a handful of progenitor domains producing at least four distinct stratified radial units. The developments to come should benefit our understanding of amygdalar functionality. Transcriptomic dissection of neuronal types in the pallial amygdala, reclassified according to our presently corroborated radial structural amygdala model, represents a novel basis for potentially accruing understanding of amygdalar computation roles in the field of normal emotional limbic mechanisms.

## Data Availability

The datasets generated during the current study are available in the GEO repository, GSE296856.

## Supplementary Materials

The following supporting information can be downloaded at: https://www.mdpi.com/article/10.3390/biom15081160/s1, Figure S1: Quality metric analysis of adult individual Seurat objects improves quality filtering; Figure S2: Quality metrics and UMAP plots indicate integration of high quality nuclei from different samples and sex in adult Seurat object; Figure S3: Gene expression for the cortical domain markers; Figure S4: Pallial amygdalar Seurat object and proportion of samples and sexes in the dataset; Figure S5: Gene markers for pallial amygdalar dataset; Figure S6: Gene markers for the *lateral* radial domain; Figure S7: Gene markers for the *basal* radial domain; Figure S8: Pallial amygdalar object from Yu et al., (2023) [14]; Figure S9: Integrated methods for pallial amygdalar objects.
