# Supplementary material for: Transcriptomic Analysis Corroborates the New Radial Model of the Mouse Pallial Amygdala
Source: Biomolecules. 2025 Aug 13;15(8):1160. doi: 10.3390/biom15081160 (PMC12384562; doi:10.3390/biom15081160)
Supplement: Supplementary file 1 [file biomolecules-15-01160-s001.zip › biomolecules-3750064-supplementary/Supplementary material Fernandez et al. 2025 (2) (1).pdf]

**Supplementary material**

# Full title: Transcriptomic analysis corroborates the new radial model of the mouse pallial amygdala

Gloria Fernández[1], Lara López-González[1,2], Eduardo Pons-Fuster[1], Luis Puelles[1], Elena Garcia-Calero[1*]

[1] Departamento de Anatomía Humana y Psicobiología, Facultad de Medicina, Universidad de Murcia e Instituto Murciano de Investigación Biosanitaria IMIB-Pascual Parrilla, Murcia, Spain.

[2] Instituto de Neurociencias, Consejo Superior de Investigaciones Científicas y Universidad Miguel Hernández, Sant Joan d'Alacant, Spain (present address).

*Corresponding author:

e-mail:ecalero@um.es

https://orcid.org/0000-0002-7184-9584

## Figure S1

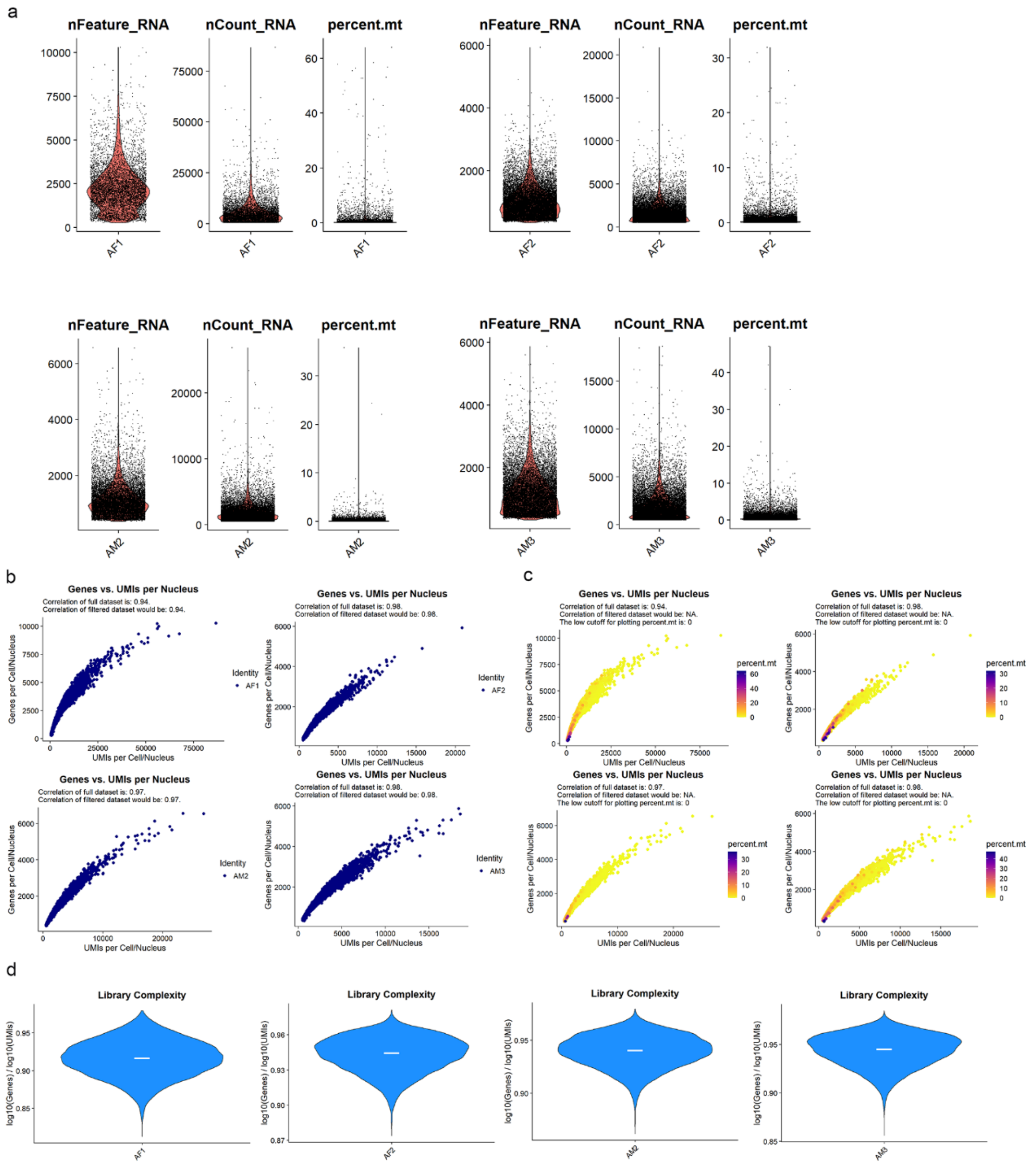


**Figure S1.** Quality metric analysis of adult individual Seurat objects improves quality filtering. (**a**) Violin plots showing features (genes) per nucleus, counts (UMIs) per nucleus and mitochondrial percentage per nucleus in each adult sample (AF1, AF2, AM2, AM3). (**b**) Scatter plots showing correlations between genes and UMIs per nucleus split by sample. (**c**) Scatter plots showing correlations between genes and UMIs per nucleus split

by sample with mitochondrial percentage as metagradient split by sample. (**d**) Libraries complexity split by sample.

# Figure S2

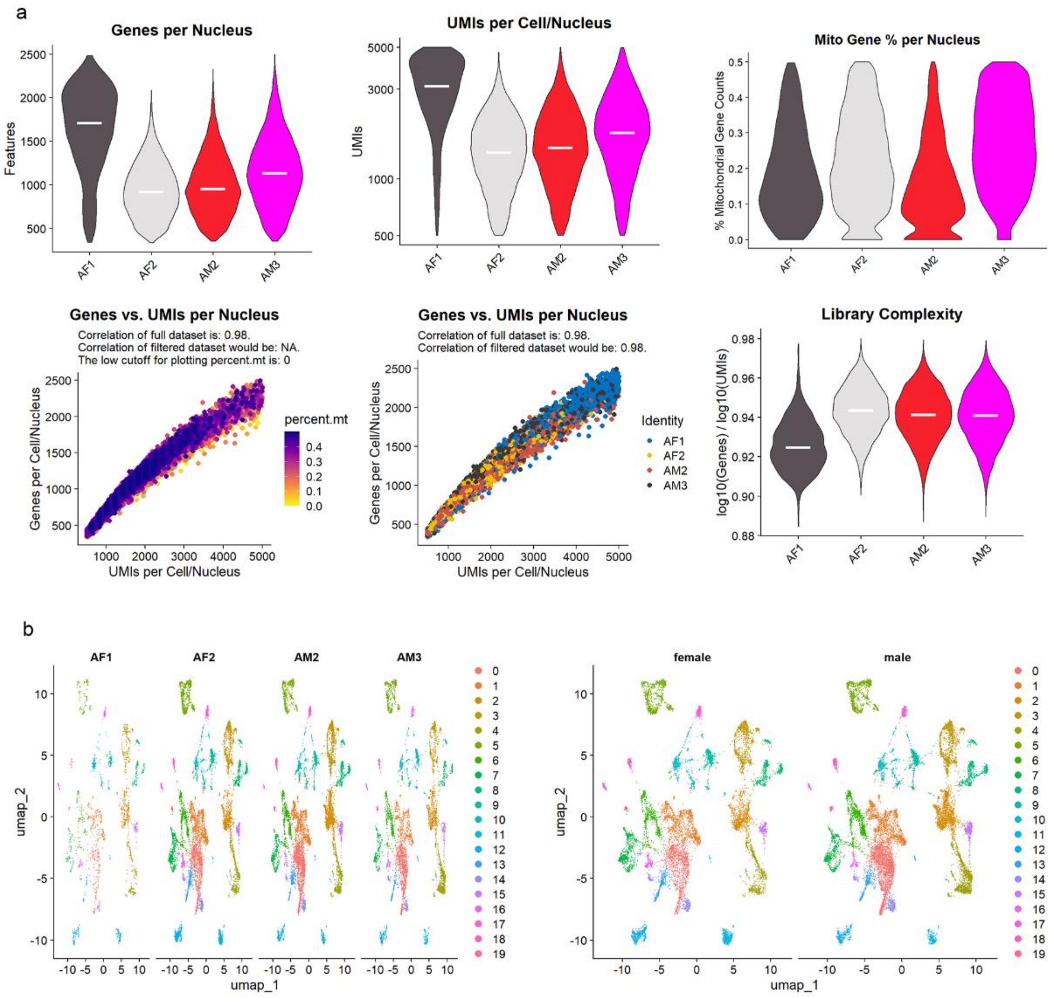


**Figure S2.** Quality metrics and UMAP plots indicate integration of high quality nuclei from different samples and sex in adult Seurat object. (**a**)Violin plots showing features (genes) per nucleus, counts (UMIs) per nucleus and mitochondrial percentage per nucleus split by sample in adult Seurat object (merge of AF1, AF2, AM2, AM3). Scatter plots show correlations between genes and UMIs per nucleus split by sample in adult Seurat object. Correlations between genes and UMIs per nucleus group by sample with mitochondrial percentage as metagradient group by sample in adult Seurat object. Libraries complexity split by sample in adult Seurat object. (**b**) UMAP plots of the adult Seurat object split by samples and by sexes.

## Figure S3

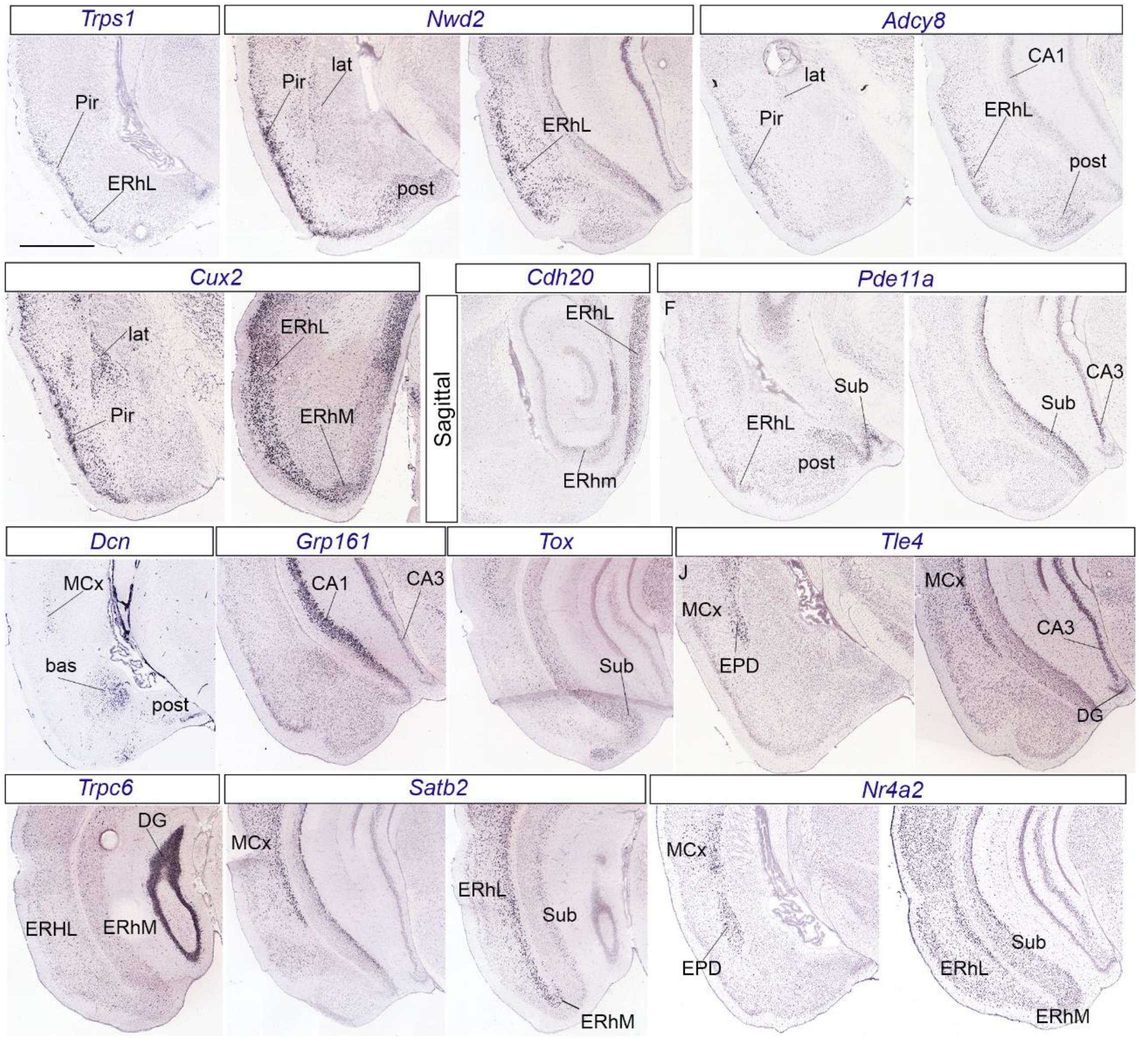


**Figure S3.** Gene expression for the cortical domain markers. *In situ* hybridization for cortical gene markers downloaded from AMBA. *Trps1, Nwd2, Adcy8, Cux2, Cdh20, Pde11a, Dcn, Grp161, Tox, Tle4, Trpc6, Satb2, Nr4a2*. Coronal plane except for *Cdh20*. Scale bar 1200 µm. ACx, allocortical ring; CA1, CA1 field; CA3, CA3 field; dentate gyrus, DG; ERhL, lateral entorhinal cortex; ERhM, medial entorhinal cortex; EPD, dorsal endopiriform nucleus; lat, *lateral* radial domain; MCx, mesocortex; MeA, medial amygdala; Pir, piriform cortex.

## Figure S4

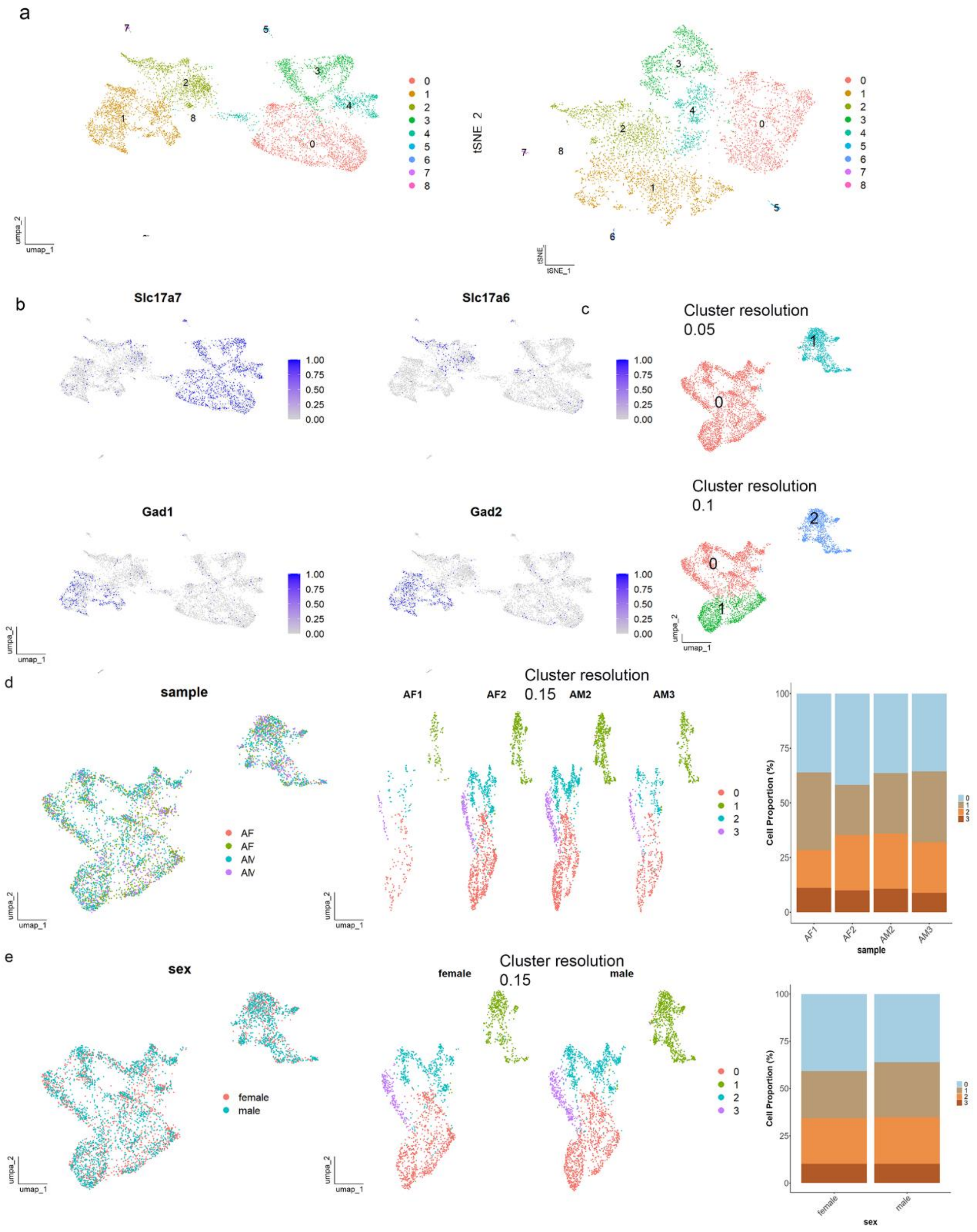


**Figure S4.** Pallial amygdalar Seurat object and proportion of samples and sexes in the dataset. (**a**) UMAP and tSNE plots of the APall clusters subsetted from the main adult Seurat object. (**b**) UMAP plots for *Slc17a7*, *Slc17a6*, *Gad1* and *Gad2* of the APall object,

showing the presence of GABAergic cells in this UMAP plots. (**c**) UMAP plot of APall clusters after removal GABAergic cells and reclusterization, cluster resolution 0.05. UMAP plot of APall clusters after removal GABAergic cells and reclusterization, cluster resolution 0.1. (**d**) UMAP plots and barplot showing four adult samples present in the four clusters in the pallial amygdalar object at cluster resolution 0.15. (**e**) UMAP plots and barplot showing both sexes present in the four clusters in the pallial amygdalar object at cluster resolution 0.15.

**Figure S5**

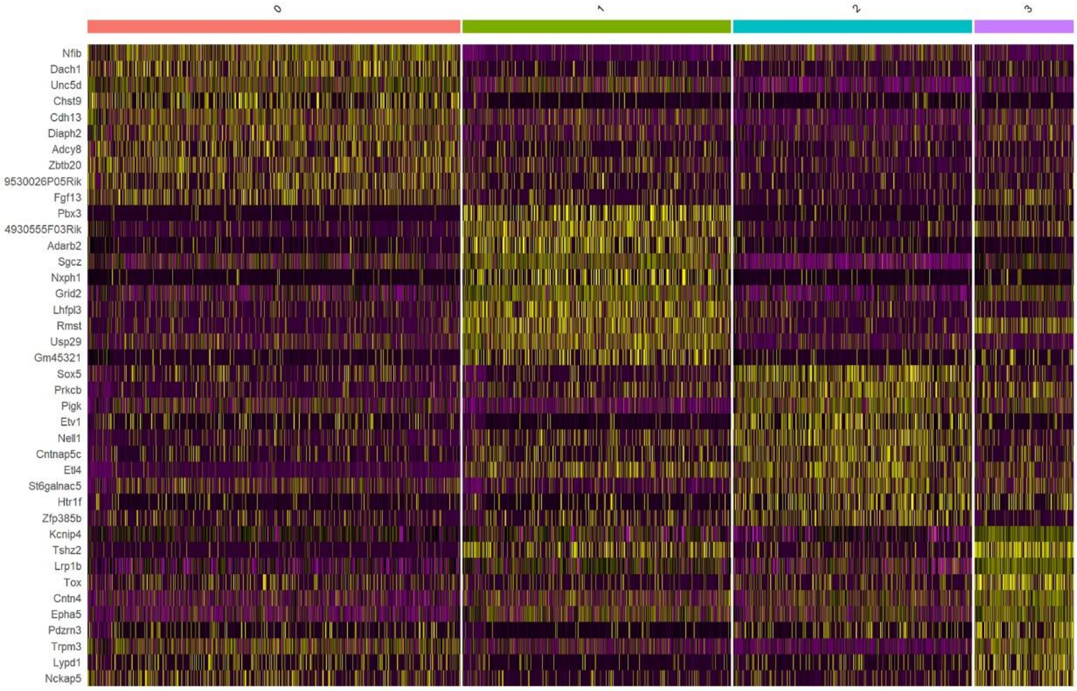


**Figure S5**. Gene markers for pallial amygdalar dataset. Heatmap of the 8 top genes differentially expressed between the four clusters in pallial amygdalar object at resolution 0.15

## Figure S6

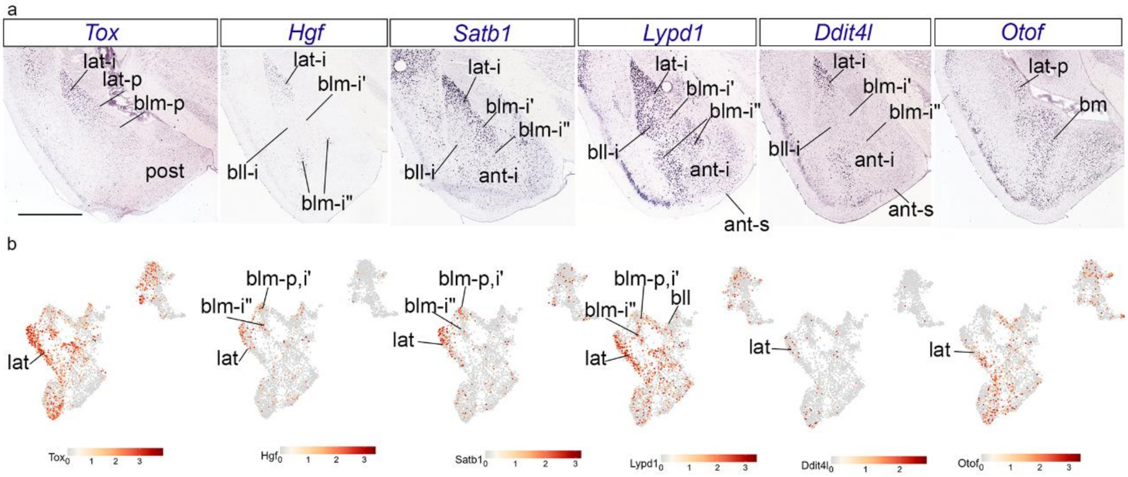


**Figure S6.** Gene markers for the *lateral* radial domain. (**a**) *In situ* hybridization for *lateral* gene markers downloaded from AMBA: *Tox, Hgf, Satb1, Lypd1, Ddit4l.* (**b**) UMAP plots for the genes *Tox, Hgf, Satb1, Lypd1, Ddit4l* in the pallial amygdalar object. Coronal plane. Scale bar 1200 µm. blm-i', blm-i", basolatero-medial radial subdomain, intermediate stratum; blm-p, *basolatero-medial* radial subdomain, periventricular stratum; bm, *basomedial* radial subdomain; lat-i, *lateral* radial domain, intermediate stratum; lat-p, *lateral* radial domain, periventricular stratum.

## Figure S7

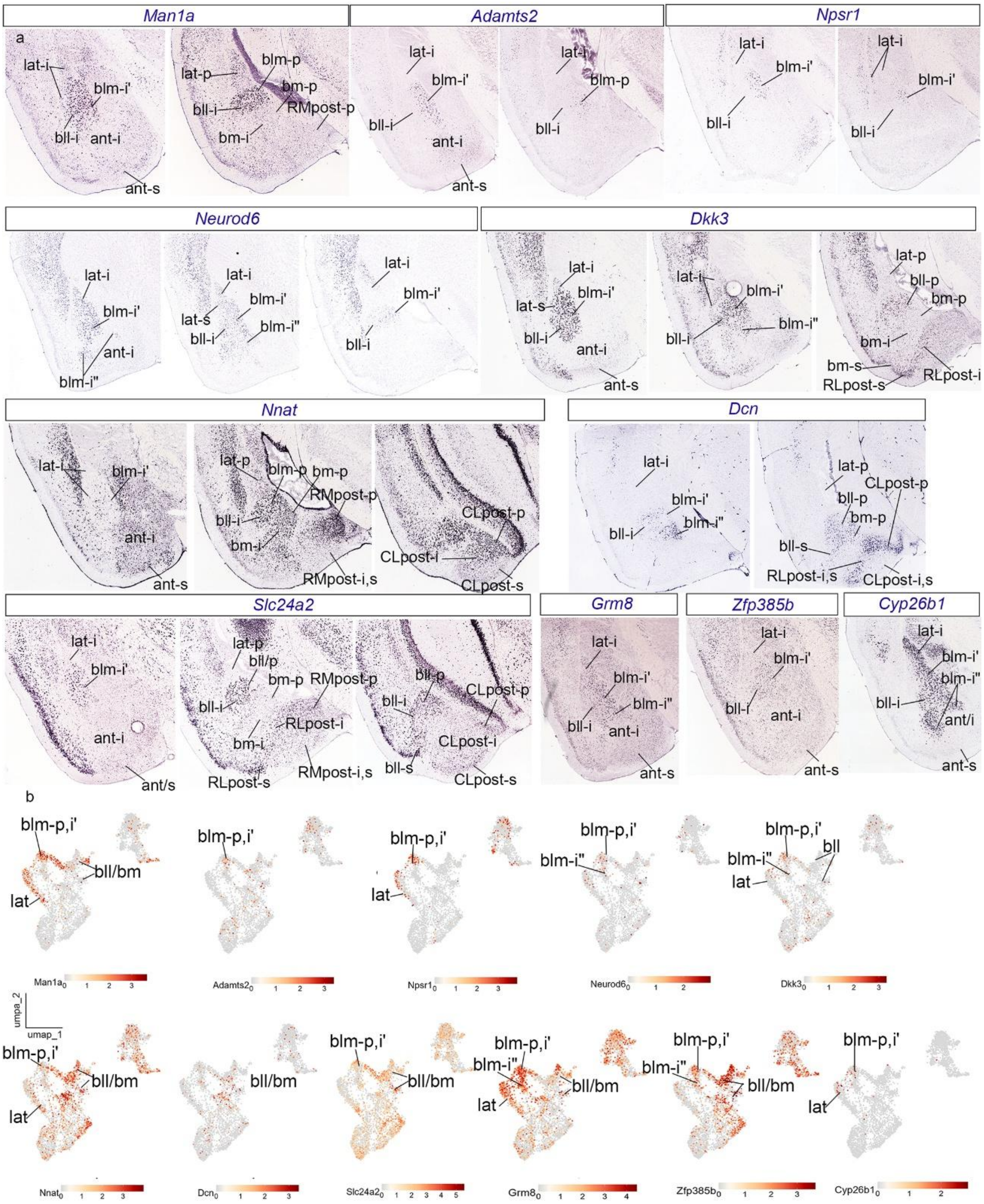


**Figure S7.** Gene markers for the *basal* radial domain. (**a**) *In situ* hybridization for *basal* gene markers downloaded from AMBA: *Man1a, Adamts2, Npsr1, Neurod6, Dkk3, Nnat, Dcn, Slc24a2, Grm8, Zfp385b, Cyb26b1*. (**b**) UMAP plots for the genes *Man1a, Adamts2, Npsr1, Neurod6, Dkk3, Nnat, Dcn, Slc24a2, Grm8, Zfp385b, Cyb26b1* in pallial

amygdalar object. Coronal plane. Scale bar 1200 μm. blm-i, *basolatero-medial* radial subdomain, intermediate stratum; blm-p, *basolatero-medial* radial subdomain, periventricular stratum; bm, *basomedial* radial subdomain; lat-i, *lateral* radial domain, intermediate stratum; lat-p, *lateral* radial domain, periventricular stratum.

## Figure S8

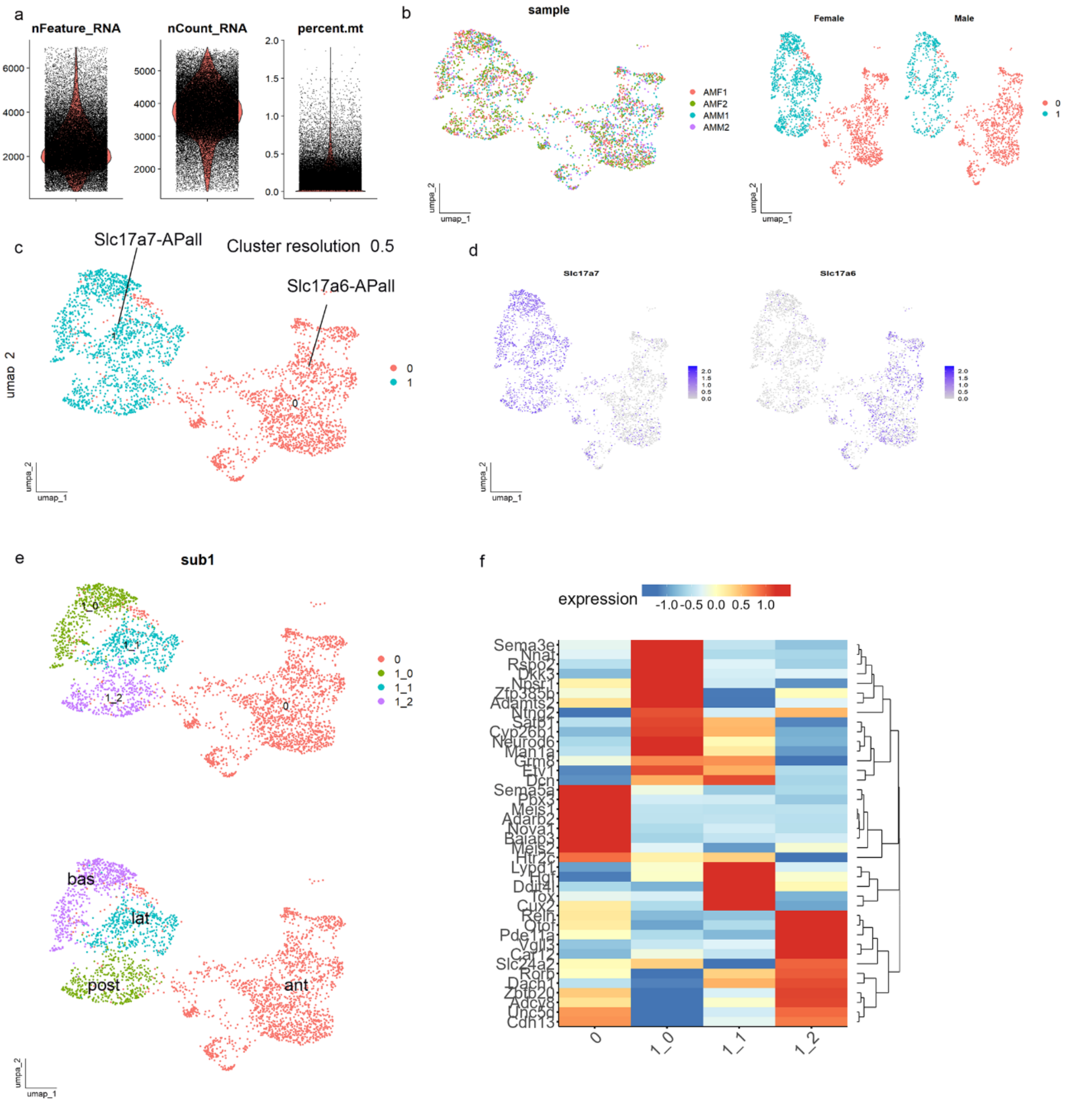


**Figure S8**. Pallial amygdalar object from Yu et al. [14]). (**a**) Violin plots showing features (genes) per nucleus, counts (UMIs) per nucleus and mitochondrial percentage per nucleus of amygdalar data set from Yu et al. [14]. (**b**) UMAP of amygdalar data set from Yu et al. [14] processed, group by sample and split by sex. (**c**) UMAP of amygdalar data set from Yu et al. [14] processed at cluster resolution 0.05. (**d**) UMAP for *Slc17a7* and *Slc17a6* in Yu et al. (2023) amygdalar dataset processed. (**e**) *Slc17a7*-APall (cluster 1) subclustered, subcluster resolution 0.12, cluster 1 is subdivided into 3 subclusters: 1_0, 1_1, 1_2. (**f**)

Heatmap of markers for pallial amygdalar domains differentially expressed between clusters and subclusters with gene hierarchical clustering of amygdalar data set from Yu et al. [14] processed.

## Figure S9

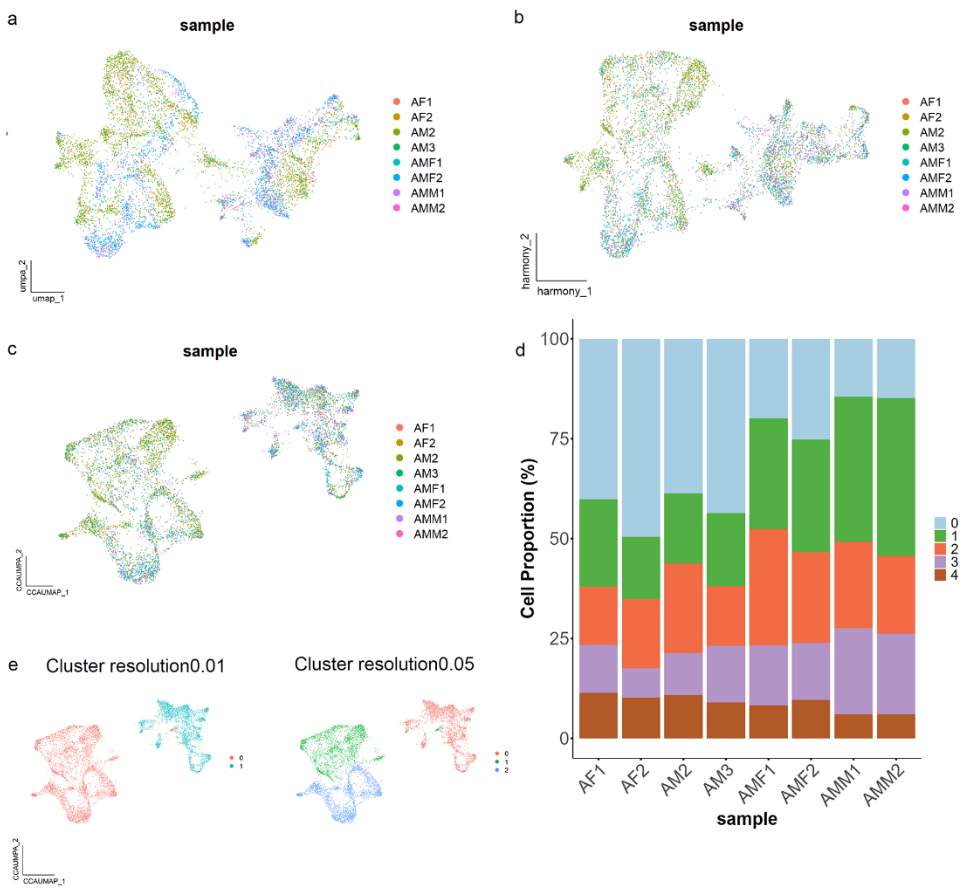


**Figure S9**. Integrated methods for pallial amygdalar objects. (**a**) UMAP plot of merge pallial amygdalar (our own results and Yu et al. (2023) dataset group by sample. (**b**) UMAP plot "Harmony" of the integrated pallial amygdalar object, group by sample. (**c**) UMAP plot "CCA" of the integrated pallial amygdalar object, group by sample. (**d**) Barplot of CCA clusters split by samples. (**e**) UMAP of the integrated pallial amygdalar object at resolution 0.01 and 0.05.
